# Concentration-Associated DNA Methylation Remodeling of Oncogenic Signaling Networks Following *Moringa oleifera* Treatment in Metastatic PC3 Prostate Cancer Cells

**DOI:** 10.3390/ijms27209144

**Published:** 2026-10-15

**Authors:** Francesca Mancuso, Cinzia Lilli, Catia Bellucci, Veronica Ceccarelli, Anna Maria Stabile, Cristiana Gambelunghe, Ludovica Pugliese, Margherita Cecchetti, Cinzia Antognelli, Tiziano Baroni

**Affiliations:** 1 Department of Medicine and Surgery, University of Perugia, 06132 Perugia, Italy; cinzia.lilli@unipg.it (C.L.); catia.bellucci@unipg.it (C.B.); veronica.ceccarelli@unipg.it (V.C.); anna.stabile@unipg.it (A.M.S.); cristiana.gambelunghe@unipg.it (C.G.); cinzia.antognelli@unipg.it (C.A.); tiziano.baroni@unipg.it (T.B.); 2 Cluster of Excellence on Cellular Stress Responses in Aging-Associated Diseases (CECAD), University of Cologne, 50931 Cologne, Germany

**Keywords:** *Moringa oleifera*, prostate cancer, PC3, DNA methylation, epigenetics, EMT, MAPK, PI3K-AKT, angiogenesis

## Abstract

PC3 prostate cancer (PCa) cells exhibit high metastatic potential, androgen independence, and activation of multiple oncogenic signaling pathways. Although *Moringa oleifera* has recognized anticancer properties, its effects on DNA methylation in aggressive PCa remain largely unexplored. Here, PC3 cells were treated with *Moringa oleifera* extract (MO) at 100 or 200 μg/mL and evaluated using functional and molecular analyses alongside genome-wide DNA methylation profiling. Both concentrations reduced AKT phosphorylation and the expression of mesenchymal and invasion-associated markers, whereas increased apoptosis was observed at 200 μg/mL MO. ERK1/2 phosphorylation differed between the two concentrations but was not significantly reduced relative to untreated controls. Methylation profiling revealed concentration-specific pathway-enrichment profiles among genes associated with differentially methylated regions (DMRs). At 100 μg/mL, hypermethylated DMR-associated genes were enriched mainly in small GTPase signaling, focal adhesion, cytoskeletal organization, axon guidance, MAPK, Rap1, and RHO signaling. At 200 μg/mL, enrichment involved cell adhesion, Wnt signaling, RAC1/CDC42 GTPase cycles, and VEGF/VEGFR2-mediated signaling. Hypomethylated DMR-associated genes showed distinct but partly overlapping enrichment profiles. In the direct comparison, DMRs with higher methylation at 100 μg/mL were associated with axon guidance, focal adhesion, actin cytoskeleton regulation, MAPK, and EPH–Ephrin signaling, whereas those with higher methylation at 200 μg/mL were associated mainly with NOTCH and RHOB/RHOC signaling. These findings identify concentration-specific DNA methylation changes in genes belonging to networks relevant to aggressive PCa, accompanied by molecular and cellular responses, without establishing a causal relationship between methylation changes and those responses.

## 1. Introduction

Prostate cancer (PCa) remains one of the leading causes of cancer-related mortality among men, and progression to castration-resistant prostate cancer (CRPC) is a major cause of therapeutic failure [1]. The human PC3 cell line is a well-established model of advanced androgen-independent PCa [2,3]. Derived from a bone metastasis, PC3 cells exhibit constitutive activation of PI3K–AKT and MAPK/ERK signaling [4,5], together with an aggressive phenotype involving epithelial–mesenchymal transition (EMT), extracellular matrix (ECM) remodeling, and high migratory and invasive capacity [6,7].

Natural compounds have attracted increasing attention as multitarget anticancer agents. *Moringa oleifera* Lam. contains bioactive metabolites with antioxidant, anti-inflammatory, antiproliferative, and pro-apoptotic properties [8,9,10,11]. We recently demonstrated that *Moringa oleifera* extract (MO) attenuates the aggressive phenotype of PC3 cells by downregulating IGF1R signaling, reducing ERK and AKT activation, modulating EMT and ECM remodeling, decreasing migration, and promoting apoptosis [12]. That study focused on the IGF-1/IGF1R axis at 100 μg/mL MO. The present study was designed as an independent follow-up to investigate whether MO treatment is associated with genome-wide DNA methylation changes and whether these changes differ between extract concentrations. PC3 cells were therefore exposed to 100 or 200 μg/mL MO, and newly generated methylation profiles were examined alongside molecular and functional data obtained within the present experimental design.

Epigenetic dysregulation, including changes in DNA methylation, histone modifications, and non-coding RNAs, contributes to PCa initiation, progression, and therapeutic response [13,14,15,16,17,18]. Aberrant DNA methylation can affect genes involved in proliferation, apoptosis, EMT, migration, invasion, angiogenesis, and treatment resistance [15,16,17,18,19,20]. Promoter hypermethylation of tumor suppressor genes, including GSTP1, APC, RASSF1A, RARB, PITX2, CCND2, and CDH1, has been associated with PCa progression and clinical outcomes [15,20,21,22]. Because epigenetic alterations are potentially reversible, they also represent attractive therapeutic targets [19].

Although *Moringa oleifera* constituents, including isothiocyanates, quercetin, and kaempferol, can modulate cancer-cell behavior, the genome-wide DNA methylation changes associated with treatment with the extract remain largely unexplored. To our knowledge, no study has investigated concentration-associated changes in genome-wide DNA methylation following *Moringa oleifera* treatment in androgen-independent PC3 cells.

Here, we combined genome-wide DNA methylation profiling with Gene Ontology (GO), Kyoto Encyclopedia of Genes and Genomes (KEGG), and Reactome enrichment analyses to characterize MO-associated methylation changes and identify pathways enriched among genes associated with differentially methylated regions (DMRs). Molecular and functional analyses were performed in parallel to assess whether these methylation changes occurred alongside cellular responses relevant to tumor progression.

## 2. Results

### 2.1. Characterization of Moringa oleifera Extract

The chemical composition of the *Moringa oleifera* ethanolic extract was previously reported by our group [12]. In the present study, GC–MS profiling was performed as a quality-control assessment to verify consistency with the previously characterized batch, rather than as a new compositional analysis. The chromatographic profile was comparable to that previously reported. The chromatogram from our previous study [12] is reproduced in Figure S1 for reference. In the previous characterization, stearic acid was the most abundant detected component (62.9% of the total normalized peak area), followed by isopropyl palmitate (18.2%), methyl oleate (7.4%), and palmitic acid (7.2%). Each remaining identified constituent accounted for less than 2% [12].

### 2.2. Apoptosis

**Figure 1:**
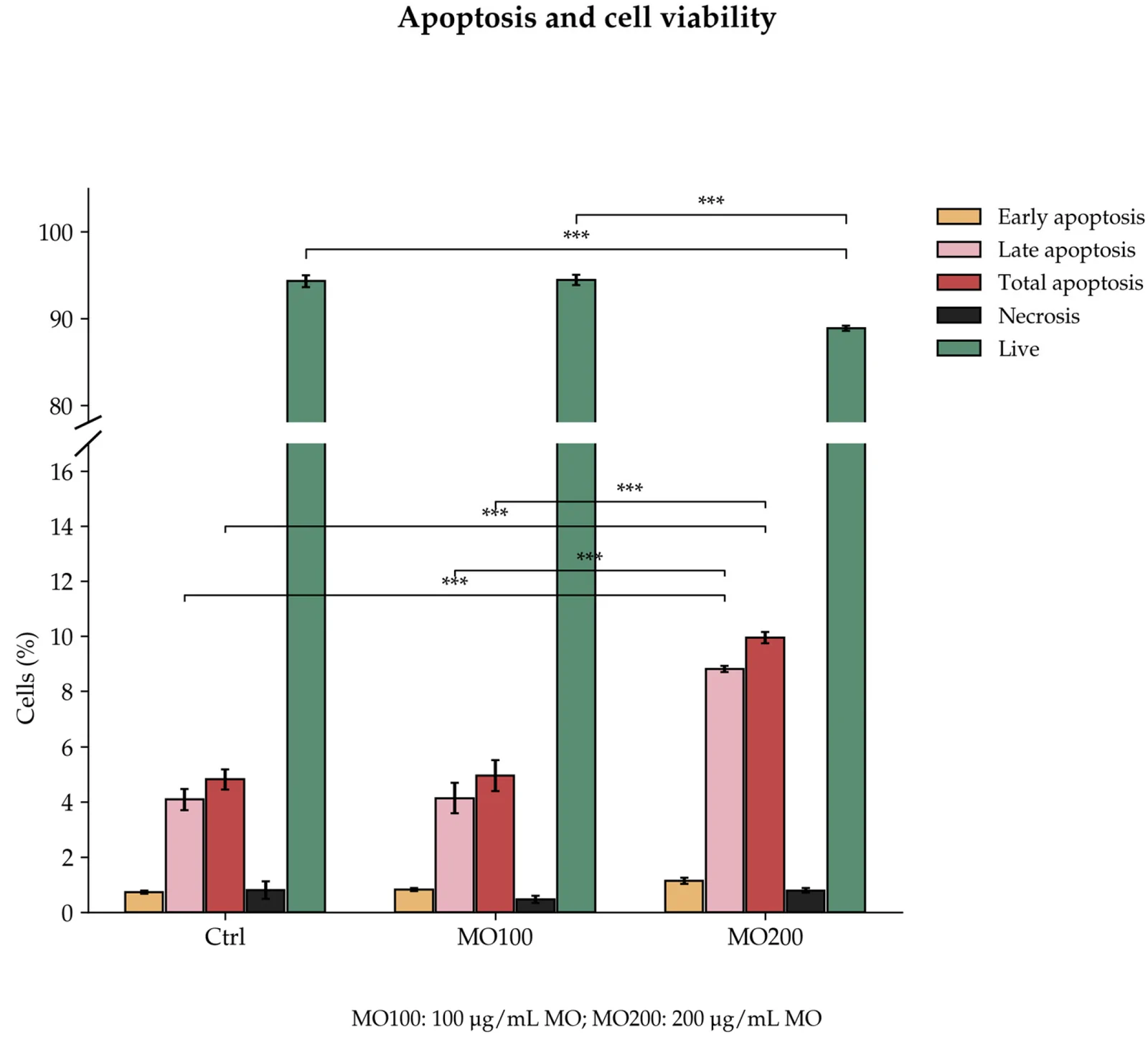
Effects of *Moringa oleifera* extract on apoptosis and cell viability in PC3 cells. Early, late, and total apoptosis, necrosis, and the percentage of viable cells were assessed after 24 h of treatment with 100 or 200 μg/mL MO. Ctrl denotes untreated control cells. Treatment with 200 μg/mL MO increased late and total apoptosis compared with both Ctrl and 100 μg/mL MO. Data are presented as the mean ± SD of three independent biological experiments. Statistical significance was assessed using two-way ANOVA followed by Tukey’s multiple comparisons test (*** *p* < 0.001).

Apoptosis was independently assessed at both 100 and 200 μg/mL *Moringa oleifera* to provide a functional reference for the comparative methylome analysis. Treatment with 200 μg/mL MO significantly increased late and total apoptosis compared with both untreated controls and cells treated with 100 μg/mL MO, whereas no significant difference was observed between 100 μg/mL MO and untreated controls (Figure 1). These experiments were performed specifically for the present study, and no data from the previously published dataset were reused.

### 2.3. Real-Time qPCR

Gene-expression analyses were independently performed at 100 and 200 μg/mL *Moringa oleifera* to complement the methylome analysis. Several endpoints previously examined at 100 μg/mL [12] were reassessed in new experiments to provide molecular context for the comparison between the two concentrations.

**Figure 2:**
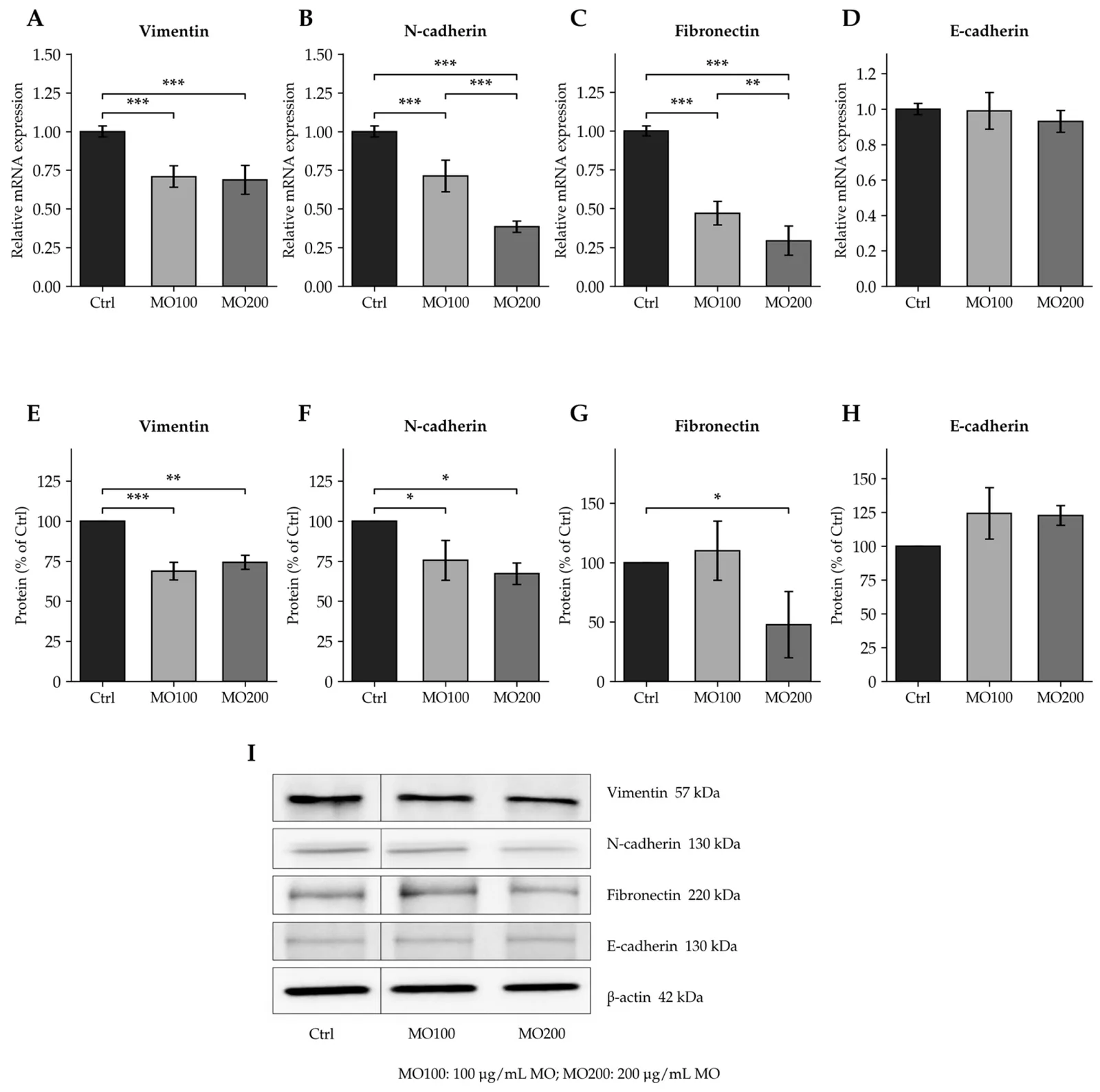
Effects of *Moringa oleifera* extract on EMT-related markers in PC3 cells. RT-qPCR analysis of vimentin (**A**), N-cadherin (**B**), fibronectin (**C**), and E-cadherin (**D**). Western blot densitometric analysis of vimentin (**E**), N-cadherin (**F**), fibronectin (**G**), and E-cadherin (**H**), normalized to β-actin. Representative immunoblots are shown in panel (**I**). Cells were left untreated (Ctrl) or treated with 100 or 200 μg/mL MO for 24 h. Black vertical lines mark non-contiguous lanes from the same original blot juxtaposed for presentation; no other alteration of the immunoblot data was performed. Uncropped original blots are provided in the Supplementary Materials. Data are presented as the mean ± SD of three independent biological experiments, each performed in triplicate. Statistical significance was assessed using two-way ANOVA followed by Tukey’s multiple comparisons test (* *p* < 0.05; ** *p* < 0.01; *** *p* < 0.001). Selected exact adjusted *p*-values were fibronectin mRNA, MO200 vs. MO100, *p* = 0.007; vimentin protein, Ctrl vs. MO100, *p* = 0.0006, and Ctrl vs. MO200, *p* = 0.0012; N-cadherin protein, Ctrl vs. MO100, *p* = 0.0476, and Ctrl vs. MO200, *p* = 0.0182. MO100 and MO200 denote 100 and 200 μg/mL MO, respectively.

**Figure 3:**
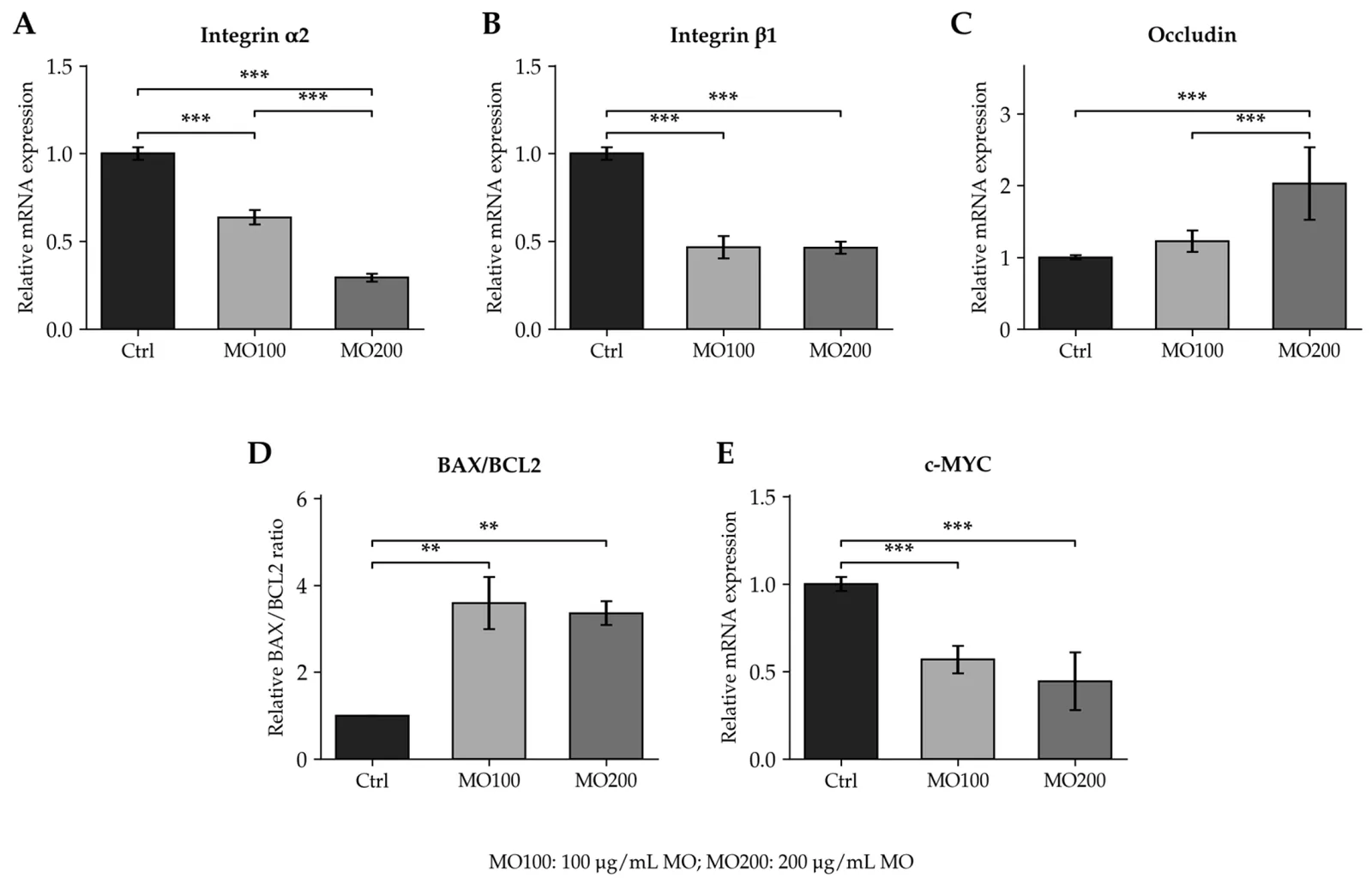
Effects of *Moringa oleifera* extract on gene expression in PC3 cells assessed by RT-qPCR. Relative expression of integrin α2 (**A**), integrin β1 (**B**), occludin (**C**), the BAX/BCL2 expression ratio (**D**), and c-MYC (**E**) after 24 h of treatment. Ctrl denotes untreated cells; MO100 and MO200 denote treatment with 100 and 200 μg/mL MO, respectively. Data are presented as the mean ± SD of three independent biological experiments, each performed in triplicate. Statistical significance was assessed using two-way ANOVA followed by Tukey’s multiple comparisons test (** *p* < 0.01; *** *p* < 0.001). Selected exact adjusted *p*-values for the BAX/BCL2 expression ratio were Ctrl vs. MO100, *p* = 0.001; Ctrl vs. MO200, *p* = 0.002.

**Figure 4:**
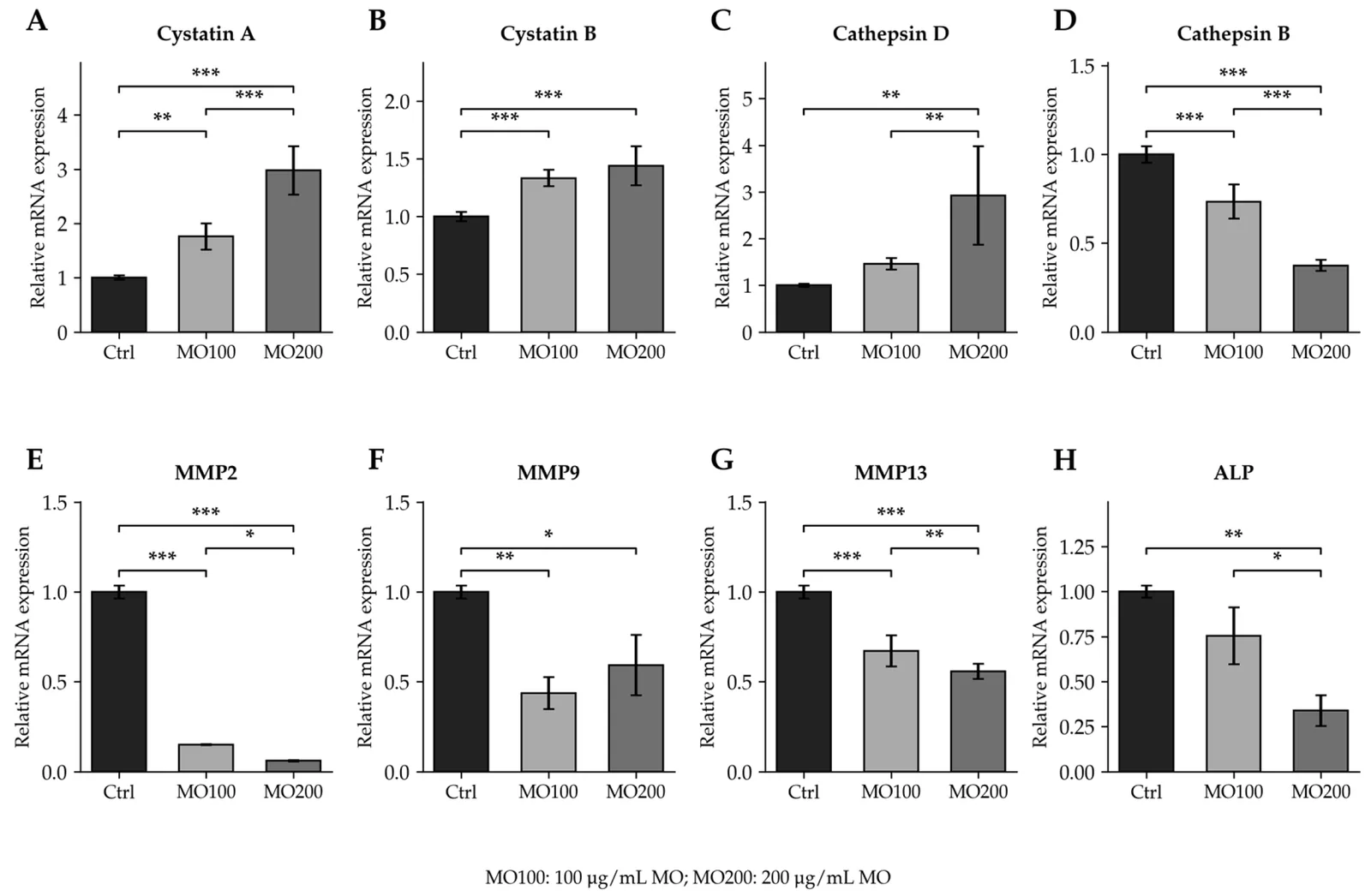
Effects of *Moringa oleifera* extract on gene expression in PC3 cells assessed by RT-qPCR. Relative expression of cystatin A (**A**), cystatin B (**B**), cathepsin D (**C**), cathepsin B (**D**), MMP2 (**E**), MMP9 (**F**), MMP13 (**G**), and alkaline phosphatase (ALP (**H**)) after 24 h of treatment. Ctrl denotes untreated cells; MO100 and MO200 denote treatment with 100 and 200 μg/mL MO, respectively. Data are presented as the mean ± SD of three independent biological experiments, each performed in triplicate. Technical replicates were averaged within each biological experiment. Statistical significance was assessed using two-way ANOVA followed by Tukey’s multiple comparisons test (* *p* < 0.05; ** *p* < 0.01; *** *p* < 0.001). Selected exact adjusted *p*-values were MMP9, Ctrl vs. MO100, *p* = 0.003, and Ctrl vs. MO200, *p* = 0.030; MMP13, MO200 vs. MO100, *p* = 0.001; cathepsin D, Ctrl vs. MO200, *p* = 0.001, and MO200 vs. MO100, *p* = 0.006; ALP, Ctrl vs. MO200, *p* = 0.002, and MO200 vs. MO100, *p* = 0.010.

Consistent with our previous observations, *Moringa oleifera* treatment reduced the expression of vimentin, N-cadherin, and fibronectin (Figure 2A–C), whereas E-cadherin expression was not significantly affected (Figure 2D). Integrin α2 and β1 expression was also reduced, while occludin expression was increased (Figure 3A–C). In addition, *Moringa oleifera* significantly increased cystatin A and cystatin B expression and reduced MMP2, MMP9, and MMP13 expression (Figure 4A,B,E–G), consistent with changes in factors associated with ECM remodeling and invasion.

c-MYC expression was significantly reduced at both *Moringa oleifera* concentrations compared with untreated controls (Figure 3E). Although the reduction was numerically greater at 200 μg/mL, the difference between the two concentrations was not statistically significant. *Moringa oleifera* treatment also significantly increased the BAX/BCL2 expression ratio at both concentrations, consistent with a pro-apoptotic molecular response (Figure 3D).

These analyses reproduced the general expression patterns previously observed at 100 μg/mL and extended the assessment to 200 μg/mL within the experimental framework of the present methylome study.

### 2.4. Western Blot Analysis

Signaling proteins were selected for Western blot analysis based on their relevance to PC3 biology and the pathways identified by methylation-enrichment analyses. MAPK signaling and MAPK-family cascades were enriched among DMR-associated genes, while Reactome analysis identified intracellular second-messenger signaling, mTOR signaling, and PIP3-mediated AKT activation. ERK1/2 and AKT phosphorylation were therefore examined as readouts of the MAPK and PI3K–AKT signaling axes. NF-κB was assessed as a complementary pro-survival and inflammatory signaling readout because of its functional interactions with these networks.

**Figure 5:**
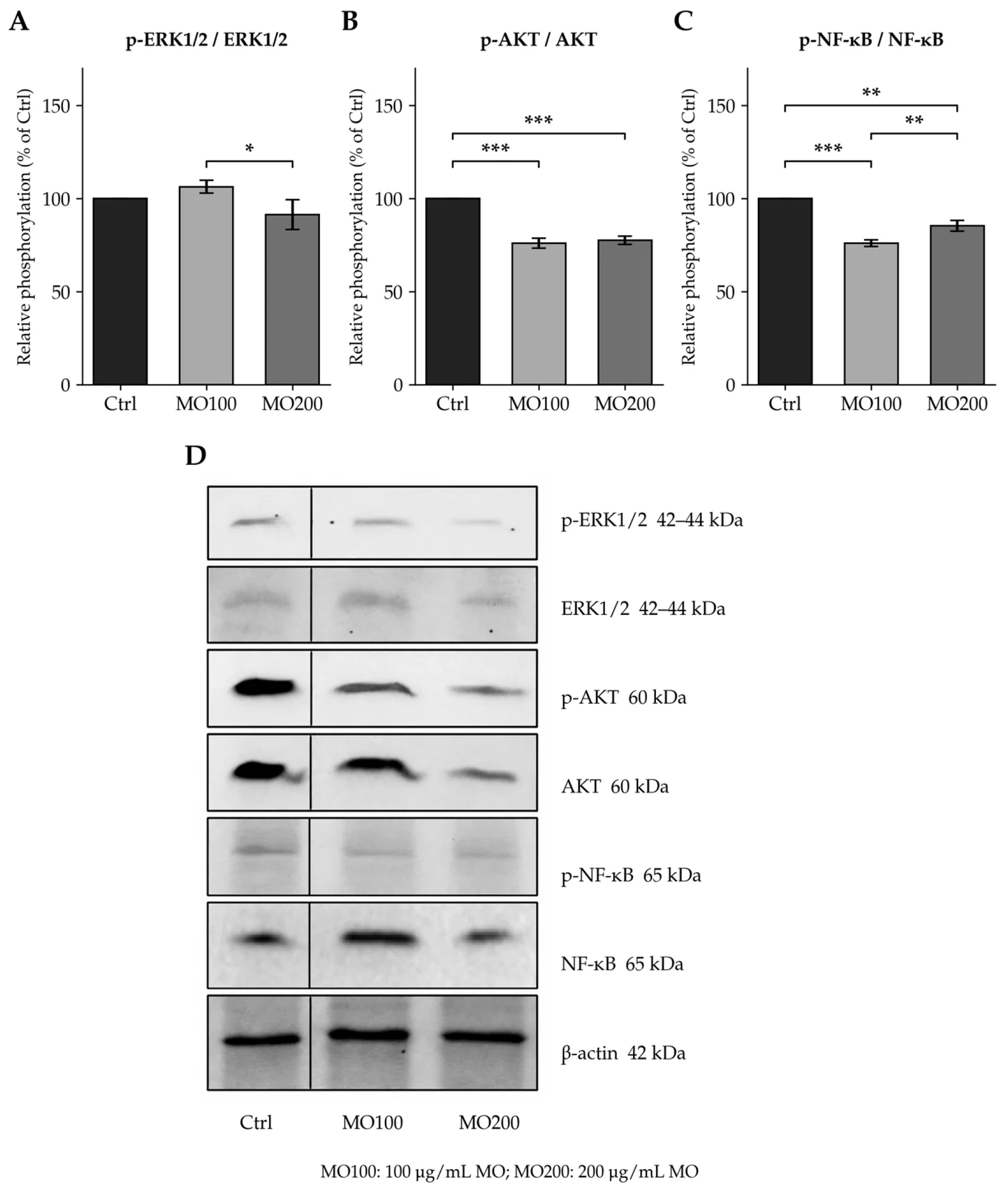
Effects of *Moringa oleifera* extract on signaling-protein phosphorylation in PC3 cells. Western blot densitometric analysis of phospho-ERK1/2/ERK1/2 (**A**), phospho-AKT/AKT (**B**), and phospho-NF-κB/NF-κB (**C**). Representative immunoblots are shown in panel (**D**). Ctrl denotes untreated cells; MO100 and MO200 denote treatment with 100 and 200 μg/mL MO for 24 h, respectively. Black vertical lines mark non-contiguous lanes from the same original blot juxtaposed for presentation; no other alteration of the immunoblot data was performed. Uncropped original blots are provided in the Supplementary Materials. Data are presented as the mean ± SD of three independent biological experiments, each performed in triplicate. Technical replicates were averaged within each biological experiment. Statistical significance was assessed using two-way ANOVA followed by Tukey’s multiple comparisons test (* *p* < 0.05; ** *p* < 0.01; *** *p* < 0.001). Selected exact adjusted *p*-values were phospho-ERK1/2/ERK1/2, MO200 vs. MO100, *p* = 0.0230; phospho-NF-κB/NF-κB, Ctrl vs. MO100, *p* = 0.0003, Ctrl vs. MO200, *p* = 0.0018, and MO200 vs. MO100, *p* = 0.0097. For phospho-AKT/AKT, adjusted *p*-values were < 0.001 for both MO concentrations compared with Ctrl.

*Moringa oleifera* treatment differentially affected ERK1/2 and AKT phosphorylation. ERK1/2 phosphorylation did not significantly differ from untreated controls at either concentration, although a significant difference was observed between MO100 and MO200 (adjusted *p* = 0.0230; Figure 5A). In contrast, AKT phosphorylation was significantly reduced at both concentrations (Figure 5B). NF-κB phosphorylation was also significantly reduced (Figure 5C). Because NF-κB was not selected as a direct methylome-derived target, this finding represents an associated molecular response rather than evidence of methylation-dependent regulation.

*Moringa oleifera* treatment significantly reduced vimentin, N-cadherin, and fibronectin protein expression, whereas E-cadherin showed a nonsignificant increase (Figure 2E–H). Representative immunoblots are shown in Figure 2I.

Together, these findings show that *Moringa oleifera* treatment is associated with changes in EMT-related proteins and signaling responses in PC3 cells.

### 2.5. Genome-Wide DNA Methylation Remodeling Associated with Moringa oleifera Treatment in PC3 Cells

Genome-wide profiling identified 12,436 hypermethylated and 5234 hypomethylated DMRs at 100 μg/mL MO relative to Ctrl, and 4397 hypermethylated and 5608 hypomethylated DMRs at 200 μg/mL MO relative to Ctrl. In the direct comparison between concentrations, 8487 DMRs showed higher methylation and 2797 showed lower methylation at 100 μg/mL relative to 200 μg/mL (Supplementary Table S2).

Pathway-enrichment analyses of DMR-associated genes revealed distinct profiles for the hypermethylated and hypomethylated gene sets at each concentration. The direct comparison also identified bidirectional methylation differences and distinct enrichment profiles, indicating concentration-associated differences in the PC3 DNA methylation landscape.

#### 2.5.1. Pathway Enrichment Among DMR-Associated Genes at 100 μg/mL *Moringa oleifera*

**Figure 6:**
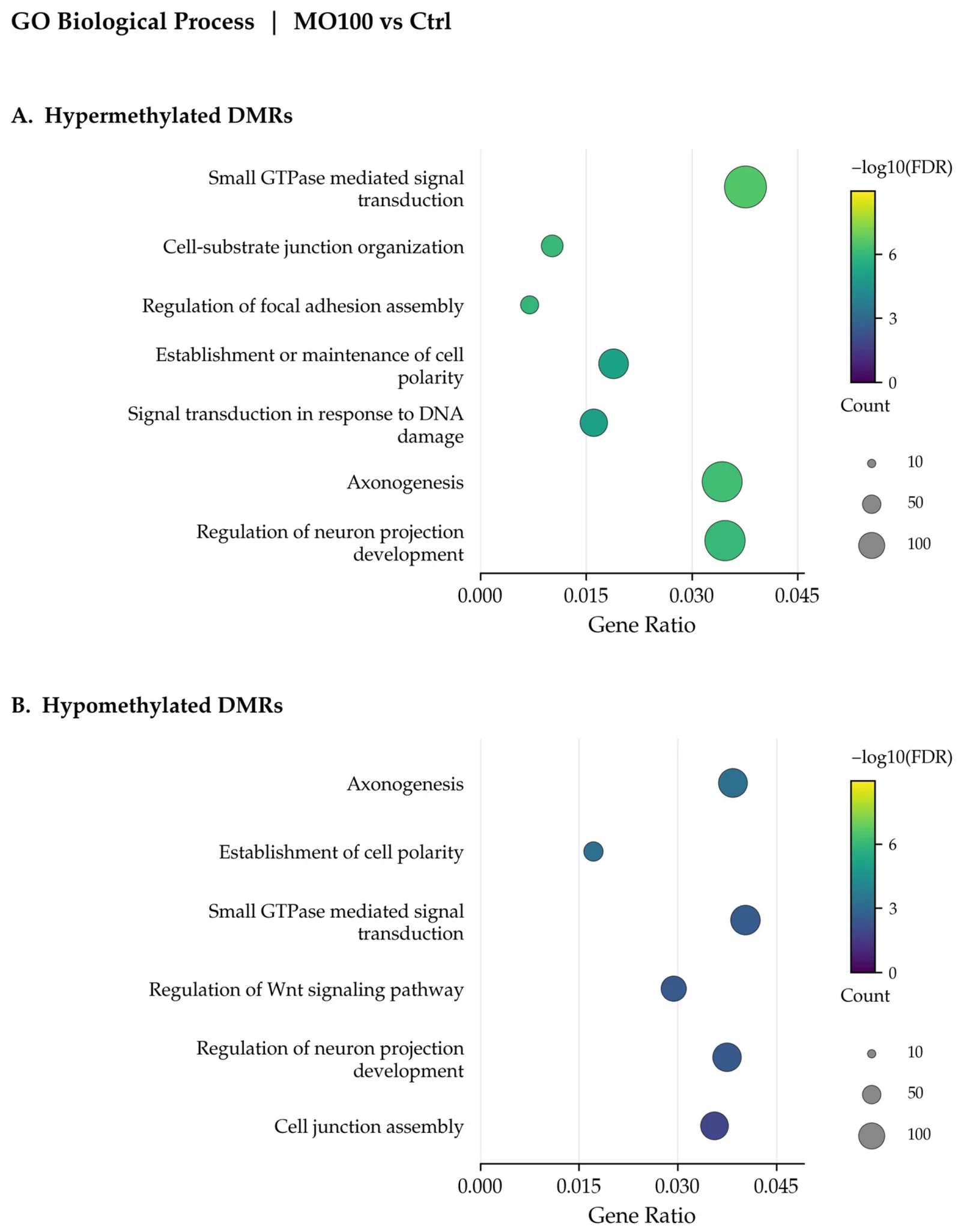
GO Biological Process enrichment analysis of DMR-associated genes in PC3 cells treated with 100 μg/mL *Moringa oleifera* extract compared with untreated controls. Selected significantly enriched terms are shown for genes associated with hypermethylated DMRs (**A**), higher methylation in MO100 relative to Ctrl and hypomethylated DMRs (**B**), lower methylation in MO100 relative to Ctrl. The *x*-axis shows the Gene Ratio, dot size represents the number of DMR-associated genes contributing to each term (Count), and dot color indicates −log10(FDR-adjusted *p*-value). Higher color-scale values indicate stronger statistical enrichment. Identical color and dot-area scales are used throughout all enrichment plots in the main text and Supplementary Materials. MO100 denotes treatment with 100 μg/mL MO; Ctrl denotes untreated cells.

**Figure 7:**
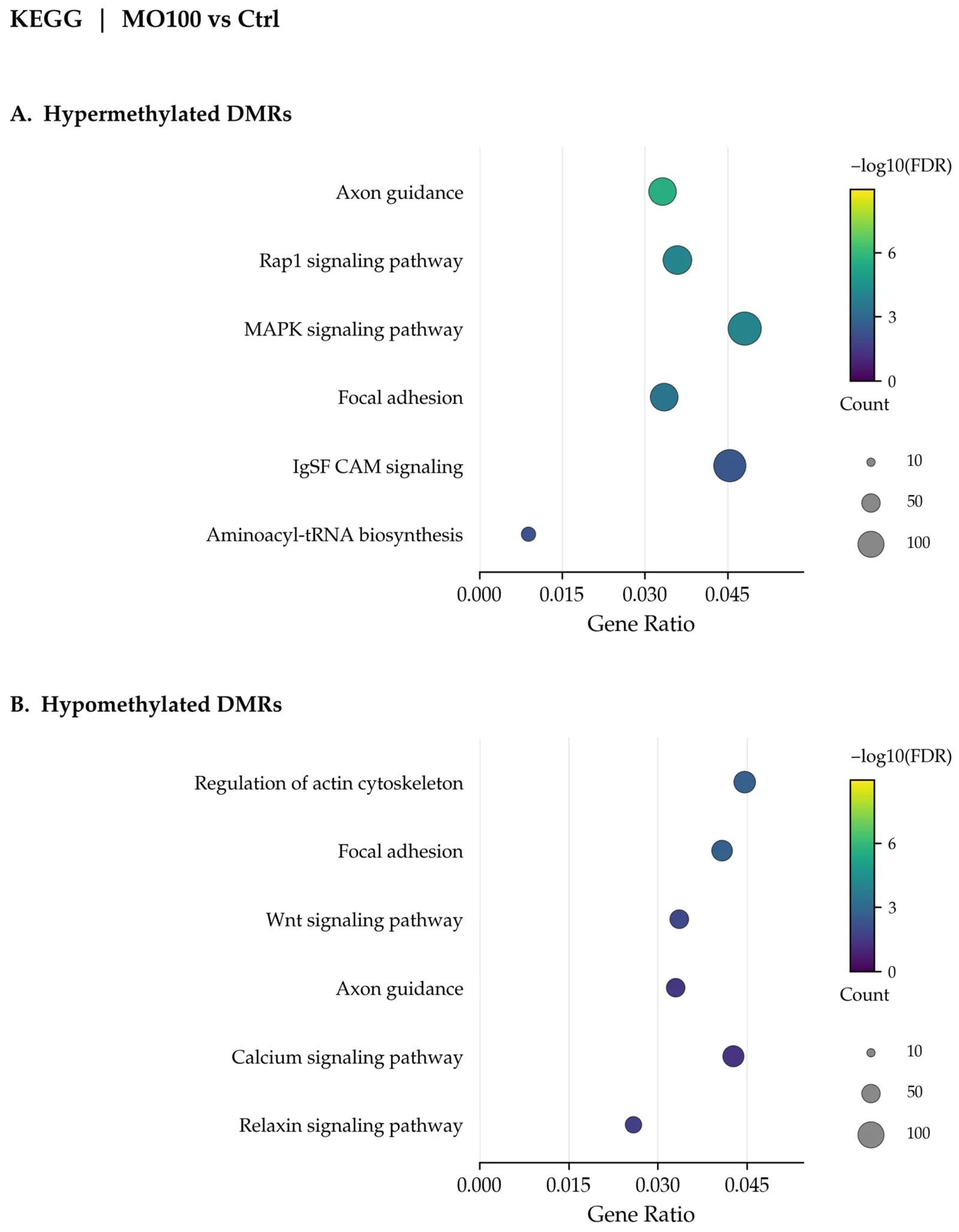
KEGG pathway enrichment analysis of DMR-associated genes in PC3 cells treated with 100 μg/mL *Moringa oleifera* extract compared with untreated controls. Selected significantly enriched pathways are shown for genes associated with hypermethylated DMRs (**A**), higher methylation in MO100 relative to Ctrl, and hypomethylated DMRs (**B**), lower methylation in MO100 relative to Ctrl. The *x*-axis shows the Gene Ratio, dot size represents the number of DMR-associated genes contributing to each pathway (Count), and dot color indicates −log10(FDR-adjusted *p*-value). Higher color-scale values indicate stronger statistical enrichment. Identical color and dot-area scales are used throughout all enrichment plots in the main text and Supplementary Materials. MO100 denotes treatment with 100 μg/mL MO; Ctrl denotes untreated cells.

At 100 μg/mL, genes associated with hypermethylated DMRs were significantly enriched in 505 GO Biological Process terms, including small GTPase-mediated signal transduction, axonogenesis, neuron projection development, cell–substrate junction organization, focal adhesion assembly, cell polarity, and protein localization to the nucleus (Figure 6A). KEGG analysis identified 53 significantly enriched pathways, including axon guidance, Rap1 and MAPK signaling, focal adhesion, IgSF CAM signaling, and aminoacyl-tRNA biosynthesis (Figure 7A). Reactome analysis identified 11 significantly enriched pathways, including the RHO GTPase cycle, mTOR signaling, intracellular second-messenger signaling, TGF-β receptor–complex signaling, semaphorin interactions, and PIP3-mediated AKT activation (Figure S2A).

Genes associated with hypomethylated DMRs were significantly enriched in 87 GO Biological Process terms, including cell polarity, axonogenesis, small GTPase signaling, regulation of Wnt signaling, neuron projection development, and cell junction assembly (Figure 6B). KEGG analysis identified 12 significantly enriched pathways, including focal adhesion, actin cytoskeleton regulation, Wnt and relaxin signaling, axon guidance, and calcium signaling (Figure 7B). Reactome analysis identified 14 significantly enriched pathways, including MAPK-family signaling cascades, collagen formation and biosynthesis, SUMOylation, and extracellular matrix organization (Figure S2B).

Overall, both hypermethylated and hypomethylated DMR-associated gene sets showed enrichment in interconnected networks related to cell motility, adhesion, and developmental signaling.

#### 2.5.2. Pathway Enrichment Among DMR-Associated Genes at 200 μg/mL *Moringa oleifera*

**Figure 8:**
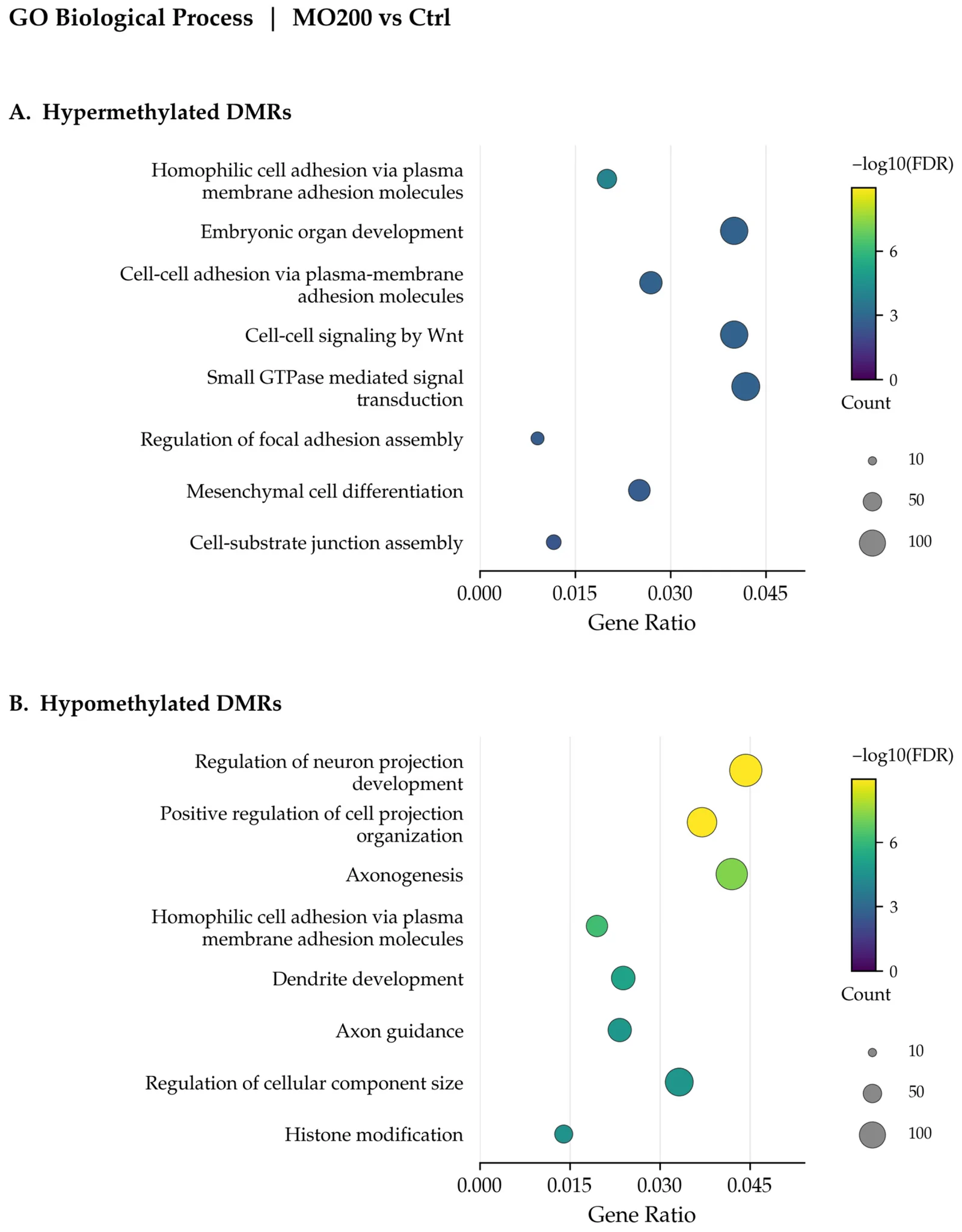
GO Biological Process enrichment analysis of DMR-associated genes in PC3 cells treated with 200 μg/mL *Moringa oleifera* extract compared with untreated controls. Selected significantly enriched terms are shown for genes associated with hypermethylated DMRs (**A**), higher methylation in MO200 relative to Ctrl and hypomethylated DMRs (**B**), lower methylation in MO200 relative to Ctrl. The *x*-axis shows the Gene Ratio, dot size represents the number of DMR-associated genes contributing to each term (Count), and dot color indicates −log10(FDR-adjusted *p*-value). Higher color-scale values indicate stronger statistical enrichment. Identical color and dot-area scales are used throughout all enrichment plots in the main text and Supplementary Materials. MO200 denotes treatment with 200 μg/mL MO; Ctrl denotes untreated cells.

**Figure 9:**
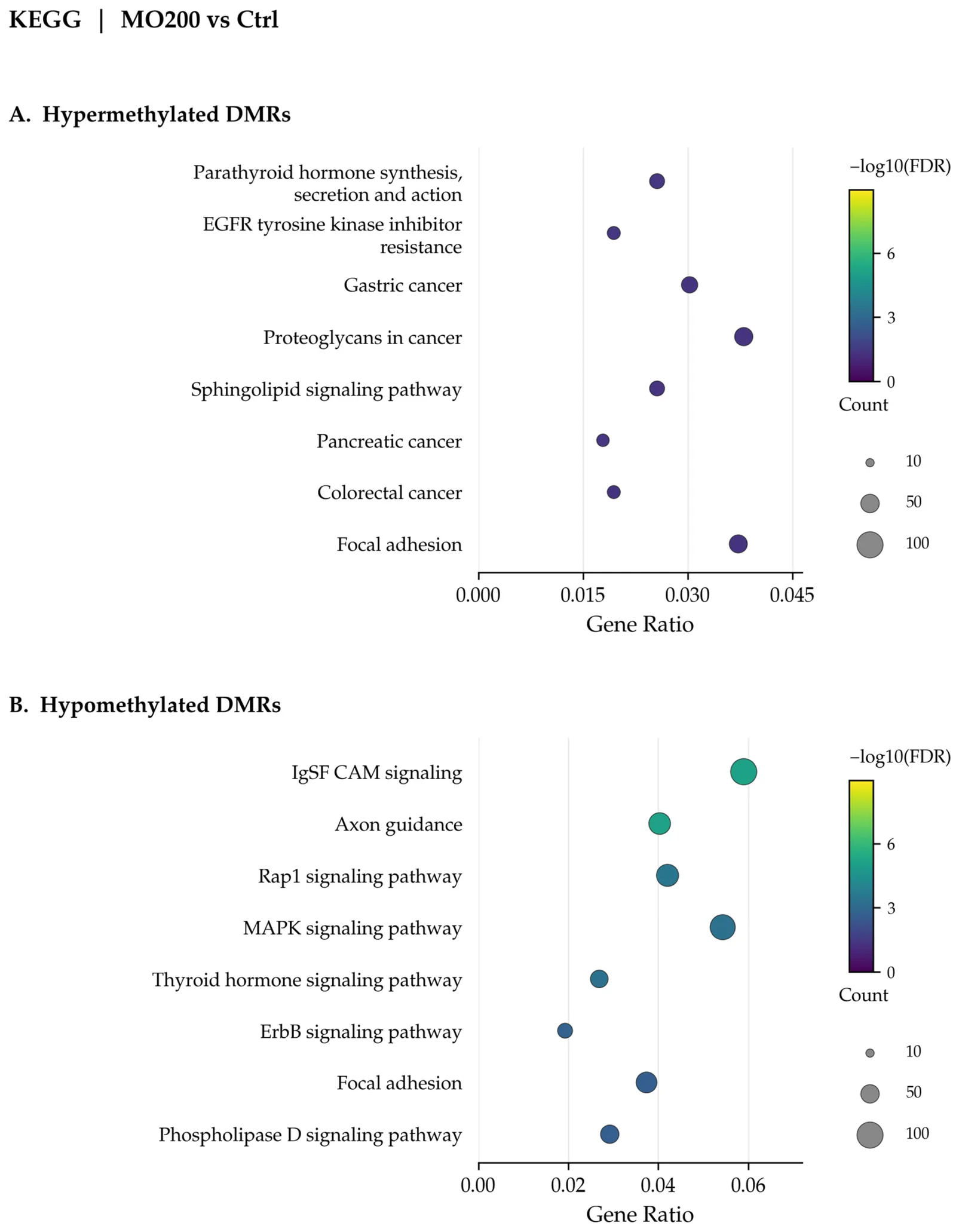
KEGG pathway enrichment analysis of DMR-associated genes in PC3 cells treated with 200 μg/mL *Moringa oleifera* extract compared with untreated controls. Selected significantly enriched pathways are shown for genes associated with hypermethylated DMRs (**A**), higher methylation in MO200 relative to Ctrl and hypomethylated DMRs (**B**), lower methylation in MO200 relative to Ctrl. The *x*-axis shows the Gene Ratio, dot size represents the number of DMR-associated genes contributing to each pathway (Count), and dot color indicates −log10(FDR-adjusted *p*-value). Higher color-scale values indicate stronger statistical enrichment. Identical color and dot-area scales are used throughout all enrichment plots in the main text and Supplementary Materials. MO200 denotes treatment with 200 μg/mL MO; Ctrl denotes untreated cells.

At 200 μg/mL, genes associated with hypermethylated DMRs were significantly enriched in 106 GO Biological Process terms. Representative terms included homophilic cell adhesion via plasma membrane adhesion molecules, embryonic organ development, cell–cell adhesion, Wnt-related signaling, small GTPase-mediated signal transduction, focal adhesion regulation, mesenchymal cell differentiation, and cell–substrate junction assembly (Figure 8A). KEGG analysis identified eight significantly enriched pathways: parathyroid hormone synthesis, secretion and action; EGFR tyrosine kinase inhibitor resistance; gastric cancer; proteoglycans in cancer; sphingolipid signaling; pancreatic cancer; colorectal cancer; and focal adhesion (Figure 9A). These disease-related annotations contain genes shared with signaling networks relevant to prostate cancer biology and do not demonstrate specific effects of MO on the indicated cancer types or mechanisms of therapeutic resistance. Reactome enrichment included the RAC1 and CDC42 GTPase cycles, VEGF signaling, DCC-mediated attractive signaling, the VEGFA–VEGFR2 pathway, and the RHO GTPase cycle (Figure S3A).

Genes associated with hypomethylated DMRs were significantly enriched in 277 GO Biological Process terms, including neuron projection development, axonogenesis, cell adhesion, dendrite development, and axon guidance (Figure 8B). KEGG analysis identified 62 significantly enriched pathways, including IgSF CAM signaling, axon guidance, Rap1, MAPK, thyroid hormone and ErbB signaling, focal adhesion, and phospholipase D signaling (Figure 9B). Reactome enrichment included intracellular second-messenger signaling, ERBB2 and RET signaling, extracellular matrix organization, NTRK signaling, MAPK-family cascades, RHO signaling, and collagen formation (Figure S3B).

Overall, hypermethylated and hypomethylated DMR-associated gene sets at 200 μg/mL showed distinct but partly overlapping enrichment profiles involving signal transduction, cell adhesion, and developmental processes.

#### 2.5.3. Direct Comparison of 100 and 200 μg/mL Reveals Distinct Concentration-Associated DNA Methylation Profiles

**Figure 10:**
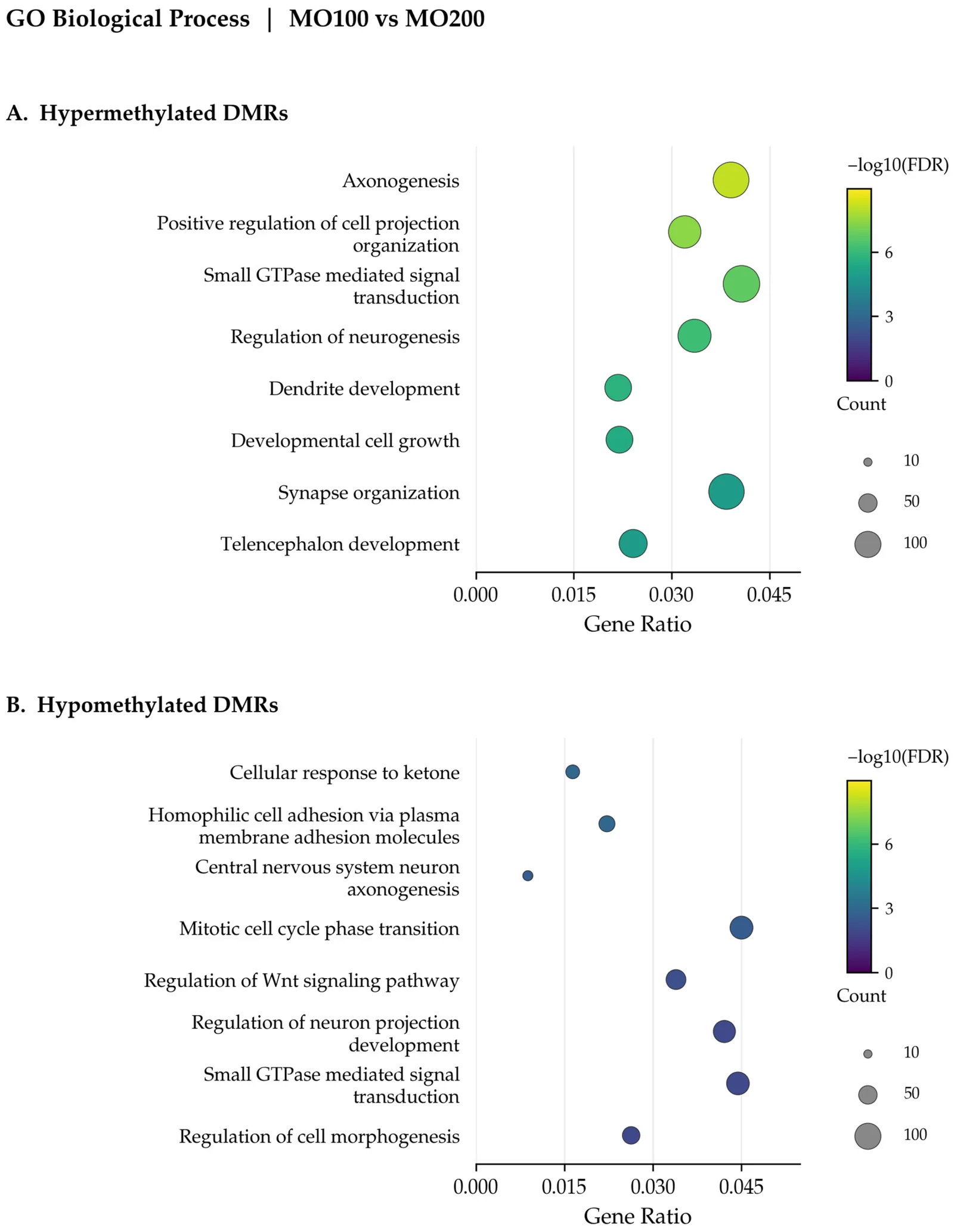
GO Biological Process enrichment analysis of DMR-associated genes in the direct comparison between PC3 cells treated with 100 and 200 μg/mL *Moringa oleifera* extract. Selected significantly enriched terms are shown for genes associated with hypermethylated DMRs (**A**), higher methylation at 100 μg/mL relative to 200 μg/mL and hypomethylated DMRs (**B**), lower methylation at 100 μg/mL relative to 200 μg/mL. The *x*-axis shows the Gene Ratio, dot size represents the number of DMR-associated genes contributing to each term (Count), and dot color indicates −log10(FDR-adjusted *p*-value). Higher color-scale values indicate stronger statistical enrichment. Identical color and dot-area scales are used throughout all enrichment plots in the main text and Supplementary Materials. MO100 denotes treatment with 100 μg/mL; MO200 denotes treatment with 200 μg/mL.

The direct comparison identified DMRs showing both higher and lower methylation at 100 μg/mL relative to 200 μg/mL. GO analysis identified 250 significantly enriched Biological Process terms among genes associated with DMRs showing higher methylation at 100 μg/mL, compared with 34 terms among genes associated with DMRs showing lower methylation at this concentration. The former included axonogenesis, positive regulation of cell projection organization, small GTPase-mediated signal transduction, regulation of neurogenesis, dendrite development, developmental cell growth, synapse organization, and telencephalon development (Figure 10A). The latter included cellular response to ketones, homophilic cell adhesion, central nervous system neuron axonogenesis, mitotic cell-cycle phase transition, regulation of Wnt signaling, small GTPase signaling, and regulation of cell morphogenesis (Figure 10B).

**Figure 11:**
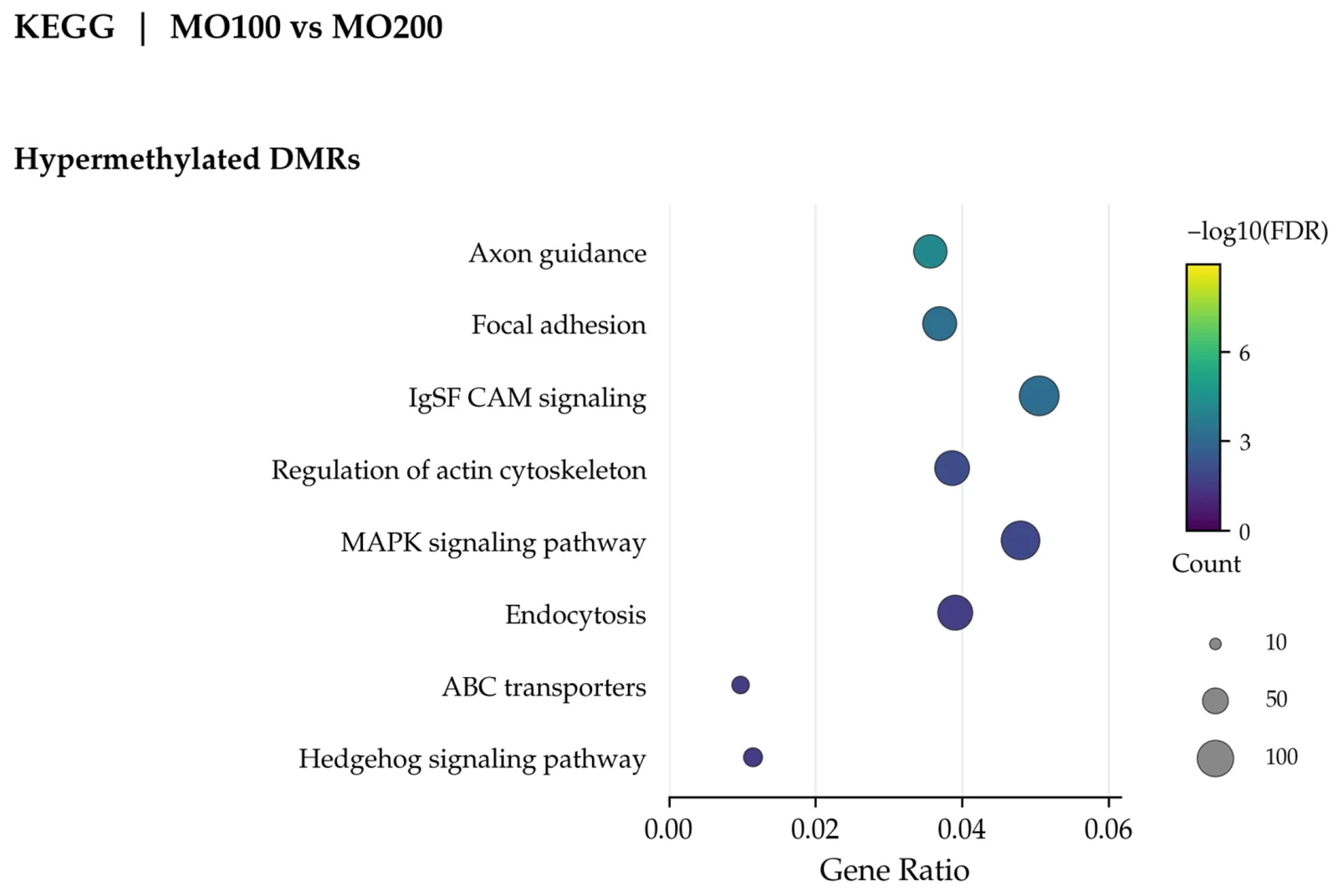
KEGG pathway enrichment analysis of DMR-associated genes in the direct comparison between PC3 cells treated with 100 and 200 μg/mL *Moringa oleifera* extract. Selected significantly enriched pathways are shown for genes associated with hypermethylated DMRs, corresponding to higher methylation at 100 μg/mL relative to 200 μg/mL. The *x*-axis shows the Gene Ratio, dot size represents the number of DMR-associated genes contributing to each pathway (Count), and dot color indicates −log10(FDR-adjusted *p*-value). Higher color-scale values indicate stronger statistical enrichment. Identical color and dot-area scales are used throughout all enrichment plots in the main text and Supplementary Materials. MO100 denotes treatment with 100 μg/mL; MO200 denotes treatment with 200 μg/mL. No significantly enriched KEGG pathways were identified among genes associated with DMRs showing lower methylation at 100 μg/mL relative to 200 μg/mL.

KEGG analysis identified 22 significantly enriched pathways among genes associated with DMRs showing higher methylation at 100 μg/mL relative to 200 μg/mL. These included axon guidance, focal adhesion, IgSF CAM signaling, actin cytoskeleton regulation, MAPK signaling, endocytosis, ABC transporters, and Hedgehog signaling (Figure 11). No significantly enriched KEGG pathways were identified among genes associated with DMRs showing lower methylation at 100 μg/mL relative to 200 μg/mL.

Reactome analysis identified 28 significantly enriched pathways among genes associated with DMRs showing higher methylation at 100 μg/mL relative to 200 μg/mL. These included EPH–Ephrin signaling, Sema3A–PAK signaling, SUMOylation-related processes, extracellular matrix proteoglycans, RHO/RAC1 GTPase cycles, non-integrin membrane–ECM interactions, MET–PTK2 signaling, and extracellular matrix organization (Figure S4A). Seven pathways were significantly enriched in the opposite direction, corresponding to higher methylation at 200 μg/mL relative to 100 μg/mL. These mainly involved RUNX3-mediated regulation of NOTCH signaling, NOTCH3/NOTCH4 signaling, and RHOB/RHOC GTPase cycles (Figure S4B).

**Figure 12:**
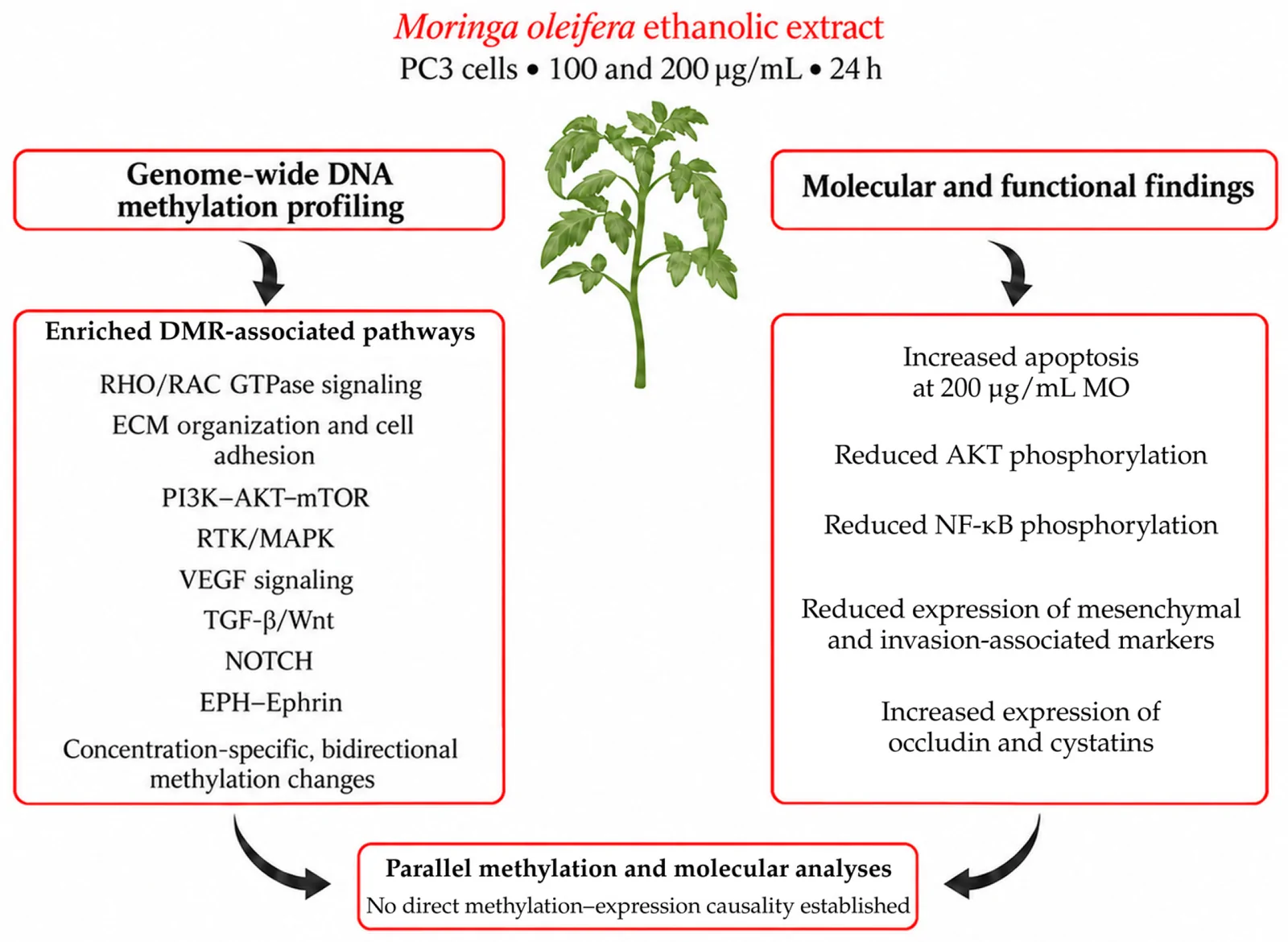
Overview of DNA methylation changes and molecular and functional findings in PC3 cells treated with *Moringa oleifera* extract. Cells were treated with 100 or 200 μg/mL MO for 24 h. Genome-wide methylation profiling identified concentration-specific, bidirectional methylation changes in DMR-associated genes enriched in pathways related to cytoskeletal organization, cell adhesion, extracellular matrix organization, and receptor-mediated signaling. Parallel analyses showed increased apoptosis at 200 μg/mL MO, reduced AKT and NF-κB phosphorylation, decreased expression of mesenchymal and invasion-associated markers, and increased expression of occludin and cystatins. These findings summarize results from parallel methylation and molecular analyses; no direct causal relationship between DNA methylation changes and molecular or cellular responses was established. Increases and decreases refer to comparisons with untreated controls. The original schematic was created in BioRender. Mancuso, F. (2026) https://BioRender.com/h49hd9q and subsequently revised using OpenAI’s ChatGPT (OpenAI, GPT-5.6; https://chatgpt.com/) image-generation tool. Final text and layout adjustments were performed using Python. All labels and scientific content were reviewed and verified by the authors.

Overall, the direct comparison revealed bidirectional methylation differences between concentrations. More enriched terms and pathways were identified among genes associated with DMRs showing higher methylation at 100 μg/mL, while a smaller set was identified in the opposite direction. An overview of the concentration-associated DNA methylation changes and the molecular and functional findings obtained from parallel analyses is provided in Figure 12.

#### 2.5.4. Integrated GO-KEGG-Reactome Interpretation

**Figure 13:**
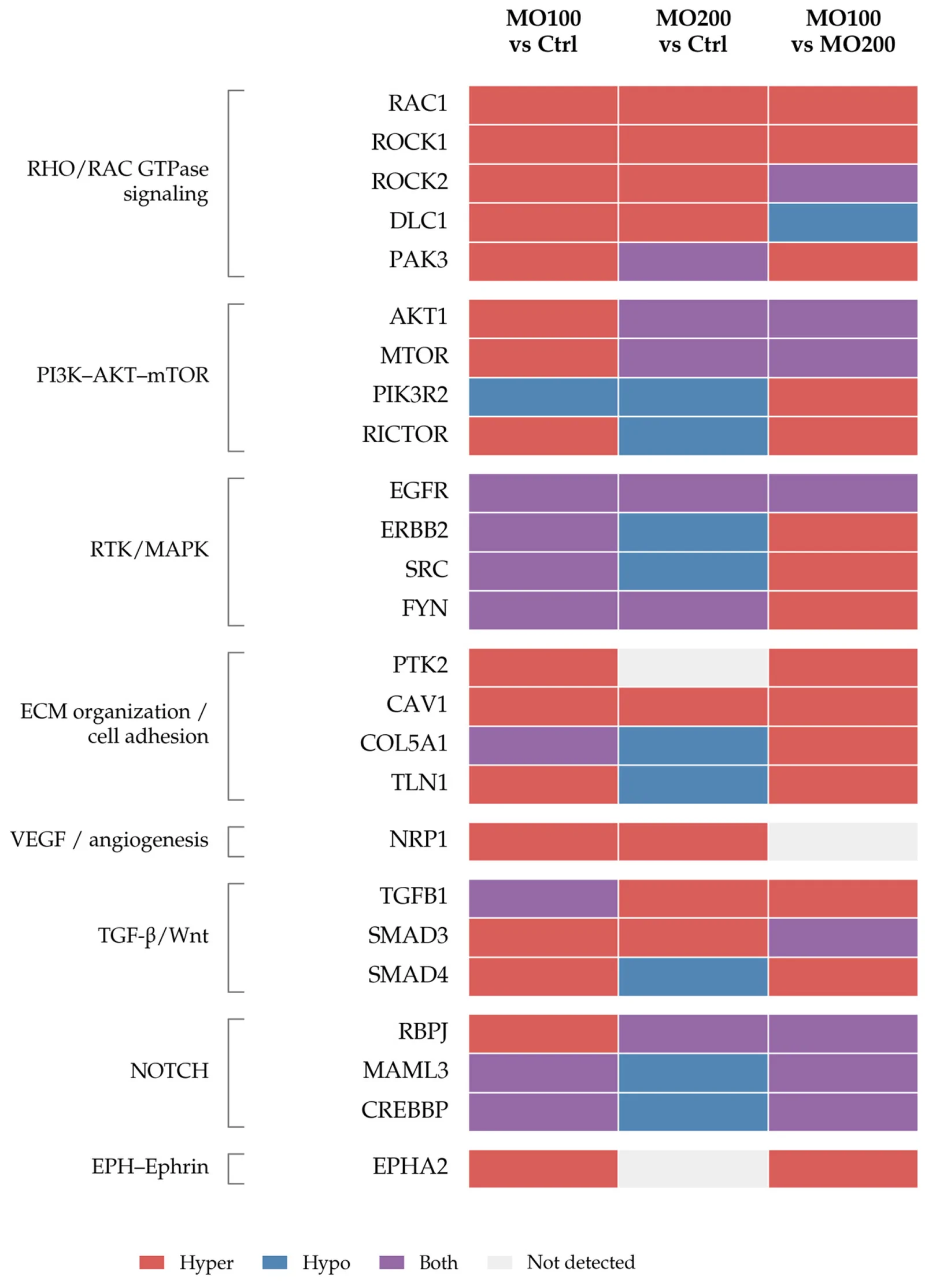
Integrated methylation landscape of candidate genes following *Moringa oleifera* treatment in PC3 cells. Representative candidate genes selected from the integrated GO, KEGG and Reactome enrichment analyses are grouped according to their major functional modules. Each cell indicates the representation of each gene in enriched DMR-associated gene sets for the indicated comparison (100 μg/mL MO vs. Ctrl, 200 μg/mL MO vs. Ctrl, and 100 μg/mL MO vs. 200 μg/mL MO), following integration of the three enrichment databases. Red indicates occurrence exclusively in enriched gene sets associated with hypermethylated DMRs, blue exclusively in those associated with hypomethylated DMRs, purple in both, and light gray absence from the integrated enrichment results. For the direct comparison between MO concentrations, hypermethylation indicates higher methylation at 100 μg/mL relative to 200 μg/mL, whereas hypomethylation indicates lower methylation at 100 μg/mL relative to 200 μg/mL. The complete list of the 86 integrated candidate genes and their database-specific annotations is provided in Table S1.

Integration of the GO, KEGG, and Reactome enrichment results identified 86 candidate genes associated with functional networks including RHO/RAC, PI3K–AKT–mTOR, receptor tyrosine kinase/MAPK, TGF-β/Wnt, NOTCH, and EPH–Ephrin signaling, as well as extracellular matrix organization, cell adhesion, and angiogenesis. Of these, 25 representative genes were selected for the directional heat map shown in Figure 13.

For each comparison, genes were classified as hypermethylated, hypomethylated, both, or not detected according to their occurrence in the enriched DMR-associated gene sets. These categories describe representation within the enrichment results; they do not establish a uniform methylation state across each gene or corresponding changes in gene expression. The complete list of 86 candidate genes and their annotations from the three databases is provided in Table S1.

## 3. Discussion

This study builds on our previous work showing that *Moringa oleifera* extract modulates IGF1R-related signaling and cellular processes relevant to prostate cancer in PC3 cells [12]. The present study addresses a distinct biological question using newly generated experimental data comparing two MO concentrations. Some phenotypic and molecular endpoints were independently reassessed to contextualize the methylome findings. The main novelty is the genome-wide characterization of DNA methylation changes and the distinct pathway-enrichment profiles observed at 100 and 200 μg/mL. To our knowledge, this is the first study to characterize genome-wide DNA methylation changes associated with a chemically characterized *Moringa oleifera* extract at two concentrations in PC3 cells. The identified DMR-associated genes were involved in networks related to cytoskeletal organization, extracellular matrix interactions, receptor-mediated signaling, angiogenesis, EMT, and metastatic behavior. These findings are relevant to PCa, in which DNA methylation abnormalities and other epigenetic alterations contribute to tumor initiation, progression, lineage plasticity, and treatment resistance [14,15,16,17,18,19,20,21,22].

PC3 cells are an androgen-independent, aggressive experimental model with phenotypic and molecular features resembling prostatic small-cell or neuroendocrine carcinoma [3,23]. Their lack of androgen receptor and prostate-specific antigen expression makes them useful for investigating androgen-independent signaling networks associated with advanced disease [4]. The enrichment profiles observed here therefore identify MO-associated methylation changes in biological networks relevant to the PC3 phenotype.

At 100 μg/mL, genes associated with hypermethylated DMRs were enriched in small GTPase signaling, focal adhesion, cytoskeletal organization, cell polarity, and axon-guidance-related processes. Rho-family GTPases and their downstream effectors coordinate actin polymerization, adhesion turnover, cell polarity, and directional migration [24]. Axon-guidance pathways are also relevant because EPH–Ephrin, semaphorin, and related guidance systems regulate tumor-cell migration, invasion, and interactions with the surrounding microenvironment [25,26]. Methylation changes involving these networks occurred alongside molecular changes consistent with reduced expression of mesenchymal and invasion-associated markers. Reduced PC3 cell migration was demonstrated in our previous study using a wound-healing assay [12]; migration and invasion were not directly assessed in the present experimental series.

The hypomethylated DMR-associated genes at 100 μg/mL showed a partly overlapping enrichment profile, including focal adhesion, actin cytoskeleton regulation, Wnt signaling, axonogenesis, MAPK-family cascades, and extracellular matrix organization. The occurrence of a pathway in both hypermethylated and hypomethylated gene sets is not necessarily contradictory: different genes or genomic regions within the same pathway may undergo methylation changes in opposite directions. Furthermore, the relationship between methylation and gene expression depends on genomic context, including promoter, enhancer, gene-body, and intergenic localization. Thus, these findings describe bidirectional methylation changes involving motility, adhesion, and developmental networks, without establishing uniform activation or inhibition of those pathways.

At 200 μg/mL, genes associated with hypermethylated DMRs showed a distinct enrichment profile involving cell–cell and cell–substrate adhesion, Wnt-related processes, small GTPase signaling, and focal adhesion regulation. Enriched KEGG pathways included cancer-associated pathways, EGFR tyrosine kinase inhibitor resistance, proteoglycans in cancer, sphingolipid signaling, and focal adhesion. These disease-related annotations reflect shared signaling genes and do not establish disease-specific effects of MO. Reactome analysis identified RAC1 and CDC42 GTPase cycles and VEGF/VEGFR2-mediated signaling. These networks connect cytoskeletal dynamics and cell–matrix interactions with processes relevant to angiogenesis and metastatic behavior. Their enrichment identifies methylation changes in associated genes, rather than directly demonstrating changes in angiogenesis or metastatic capacity.

Genes associated with hypomethylated DMRs at 200 μg/mL were also enriched in signaling, structural, and developmental networks, including Rap1, MAPK, ErbB, Hippo, VEGF, RET, and NTRK signaling, focal adhesion, and extracellular matrix organization. Several of these pathways contribute to prostate cancer progression and cellular plasticity [4,20,27,28]. The hypomethylated component therefore cannot be classified as exclusively non-oncogenic or biologically neutral. Its functional consequences remain unresolved without transcriptomic integration and locus-specific validation.

The direct comparison between MO concentrations further demonstrated distinct methylation profiles. DMRs showing higher methylation at 100 μg/mL relative to 200 μg/mL were associated with more enriched GO terms and with 22 significantly enriched KEGG pathways. These included axon guidance, focal adhesion, IgSF CAM signaling, actin cytoskeleton regulation, MAPK signaling, endocytosis, ABC transporters, and Hedgehog signaling. Reactome enrichment in the same direction included EPH–Ephrin signaling, Sema3A–PAK signaling, RHO/RAC1 GTPase cycles, extracellular matrix interactions, and MET–PTK2 signaling. These results support concentration-specific methylation profiles rather than a simple progressive increase in methylation with increasing MO concentration.

Conversely, the seven enriched Reactome pathways associated with DMRs showing higher methylation at 200 μg/mL relative to 100 μg/mL mainly involved RUNX3-mediated NOTCH regulation, NOTCH3 and NOTCH4 signaling, and RHOB and RHOC GTPase cycles. NOTCH signaling has context-dependent roles in PCa and has been associated with tumor-cell plasticity, invasion, and progression [29,30]. Individual RHO-family members may likewise have different or opposing functions depending on the cellular context. No significantly enriched KEGG pathways were identified for this methylation direction. The difference between KEGG and Reactome results may reflect differences in pathway definitions, annotation coverage, and gene-set composition.

Integration of GO, KEGG, and Reactome enrichment results identified 86 candidate genes, of which 25 representative genes were selected for the directional heat map. Several genes occurred in both hypermethylated and hypomethylated enriched gene sets, illustrating the complexity of the observed methylation patterns. The categories in Figure 13 describe each gene’s representation within these enriched DMR-associated gene sets; they do not indicate a uniform methylation state across the gene or a corresponding change in its expression. The selection was descriptive and did not involve formal assessment of network centrality or hub status.

The molecular and functional findings provided complementary biological context. MO treatment increased apoptosis at 200 μg/mL and significantly reduced AKT phosphorylation. ERK1/2 phosphorylation differed between the two MO concentrations but did not significantly differ from untreated controls at either concentration. ERK1/2 and AKT were examined because of their established relevance to PC3 biology and the enrichment of DMR-associated genes in MAPK, PI3K–AKT/mTOR, and related signaling networks. Previous studies have also reported that *Moringa oleifera* extracts or isolated fractions inhibit PC3-cell proliferation, promote apoptosis, and affect AKT-dependent signaling [11,31]. Reduced NF-κB phosphorylation provided an additional readout of the molecular response to MO. NF-κB was assessed as a complementary signaling endpoint, rather than as direct validation of a methylome-derived target.

Gene-expression analyses showed reduced expression of mesenchymal and invasion-associated markers, including vimentin, N-cadherin, fibronectin, integrins, and matrix metalloproteinases, together with increased expression of occludin and cystatins. Reduced ALP expression was consistent with our previous observations [12] and with attenuation of an osteomimetic molecular feature of PC3 cells. These changes are biologically consistent with the involvement of adhesion, extracellular matrix, and cytoskeletal networks in the enrichment analyses. However, the expression and phosphorylation measurements do not demonstrate that specific methylation changes caused these molecular responses. The lower ERK and AKT activation reported in our previous IGF-1/IGF1R-focused study at 100 μg/mL [12] should also be distinguished from the present findings, in which a significant reduction relative to controls was observed for AKT, but not ERK1/2.

Previous studies have associated Moringa-derived compounds with changes in DNA methylation and gene expression in other experimental models [32,33]. More broadly, plant-derived bioactive compounds can affect DNA methylation, histone modifications, and non-coding RNA regulation [33,34,35,36,37]. Such mechanisms may be relevant to the chemically heterogeneous *Moringa oleifera* extract used here [38,39,40,41]. Nevertheless, the present study did not assess DNA methyltransferases, TET dioxygenases, histone-modifying enzymes, or non-coding RNAs. The mechanisms underlying the observed methylation changes therefore remain unknown, and the effects cannot be attributed to an individual constituent. Studies using standardized fractions or isolated compounds will be needed to identify the active constituents and their mechanisms.

Several limitations should be considered. First, the study used a single cell line, which does not represent the biological heterogeneity of PCa. Confirmation in additional models, including DU145 cells, androgen-responsive cells, patient-derived organoids, and in vivo systems, is needed. Second, the methylome analysis included a small number of biological samples, particularly at 200 μg/mL (n = 2), limiting statistical power and the robustness of comparisons between concentrations. Larger independent sample sets are required to validate these findings.

Third, enrichment analysis is subject to potential array-related representation bias. Although the background universe included all genes associated with the analyzed DMPs, irrespective of statistical significance, no formal correction was applied for gene length, CpG density, or unequal CpG-probe representation on the MethylationEPIC v2.0 array. These factors may influence enrichment results. In addition, pathway associations do not establish transcriptional activation or repression. Integration with RNA sequencing and locus-specific methylation validation, potentially complemented by chromatin-accessibility and histone-mark analyses, will be needed to determine the functional consequences of the observed changes.

Finally, the concentrations used in vitro should not be assumed to be achievable in prostate tissue following oral administration of *Moringa oleifera*. The bioavailability, metabolism, pharmacokinetics, and tissue distribution of the extract’s constituents remain to be established. The present study identifies concentration-specific, bidirectional DNA methylation changes accompanied by molecular and cellular responses in PC3 cells. These findings provide a basis for further mechanistic investigation but do not establish methylation-mediated causality or support therapeutic use in patients.

## 4. Materials and Methods

### 4.1. Preparation of Moringa oleifera Extract

The preparation of the *Moringa oleifera* extract was performed as previously described in our earlier study [12]. Briefly, dried leaf powder was extracted by maceration in 70% (*v*/*v*) ethanol, prepared by dilution of absolute ethanol (≥99%, Cat. No. E7023; Sigma-Aldrich, St. Louis, MO, USA) with distilled water, under continuous agitation at room temperature, followed by centrifugation, filtration, and solvent evaporation. The dried residue was weighed and reconstituted to obtain a stock solution, which was diluted in culture medium to reach the desired experimental concentrations.

### 4.2. GC-MS Analysis

Chemical characterization of the extract was performed by GC-MS analysis as previously described [12]. The extract was derivatized with MSTFA and analyzed using an Agilent gas chromatography-mass spectrometry system (Agilent Technologies, Santa Clara, CA, USA). Compounds were identified by comparison with the NIST spectral library.

### 4.3. PC3 Cell Line and Culture Conditions

PC3 human prostate adenocarcinoma cells (ATCC^®^ CRL-1435™; American Type Culture Collection, Manassas, VA, USA), purchased from the Experimental Zooprophylactic Institute of Lombardia and Emilia-Romagna (Brescia, Italy), were cultured in F-12K Nut Mix medium supplemented with 10% fetal bovine serum (FBS; Gibco™, Cat. No. 10270106; Thermo Fisher Scientific, Waltham, MA, USA) and penicillin/streptomycin (1×), obtained from a 100× stock solution containing 10,000 U/mL penicillin and 10,000 μg/mL streptomycin (Gibco™, Cat. No. 15140122; Thermo Fisher Scientific, Waltham, MA, USA). Phosphate-buffered saline (PBS, pH 7.4, without calcium and magnesium; Gibco™, Cat. No. 10010023; Thermo Fisher Scientific, Waltham, MA, USA) and 0.05% Trypsin-EDTA (Gibco™, Cat. No. 25300054; Thermo Fisher Scientific, Waltham, MA, USA) were used for routine cell washing and passaging, respectively. Cells were maintained at 37 °C in a humidified atmosphere containing 5% CO_2_. Morphological changes following treatments were monitored using an inverted optical microscope.

### 4.4. Experimental Design

The concentrations selected for the present study were based on our previously published concentration-response analysis in PC3 cells [12]. The experiments reported in the present study were performed within a new experimental series designed specifically for the comparative molecular and methylome analyses described here. Data obtained in the previous publication were not reused as experimental replicates in the present analyses. In that study, *Moringa oleifera* extract produced only moderate reductions in cell viability across the concentration range of 50–300 μg/mL, while preserving substantial cellular viability and biological responsiveness. These findings identified 100 and 200 μg/mL as biologically active, non-cytotoxic concentrations suitable for investigating molecular and epigenetic effects.

For functional, molecular, and genome-wide DNA methylation analyses, PC3 cells were seeded at a density of 3 × 10^5^ cells/well in 6-well plates and allowed to attach for 48–72 h. Untreated PC3 cells served as controls, whereas treated cells were exposed to *Moringa oleifera* extract (100 or 200 μg/mL) for an additional 24 h. Following treatment, total RNA and protein extracts were prepared as previously described [12]. In parallel, genomic DNA was isolated and subjected to genome-wide DNA methylation profiling using the Infinium MethylationEPIC v2.0 BeadChip platform (Illumina, San Diego, CA, USA), enabling the assessment of genome-wide DNA methylation changes associated with the two MO concentrations.

### 4.5. Apoptosis Assay

Apoptosis was evaluated by flow cytometry using eBioscience™ Annexin V-FITC Apoptosis Detection Kit (Cat. No. 88-8005-72; Invitrogen™, Thermo Fisher Scientific, Waltham, MA, USA), as previously described [42]. This method allows discrimination between viable, early apoptotic, late apoptotic and necrotic cells.

### 4.6. Real-Time Quantitative PCR (RT-qPCR)

Total RNA was extracted using TRIzol™ Reagent (Cat. No. 15596026; Invitrogen™, Thermo Fisher Scientific, Waltham, MA, USA). RNA concentration and purity were assessed spectrophotometrically at 260 nm. cDNA was synthesized from 1 μg of total RNA using All-In-One 5X RT MasterMix with gDNA Removal (Cat. No. G592; Applied Biological Materials Inc., Richmond, BC, Canada). Quantitative PCR was performed using BlasTaq™ 2X qPCR MasterMix (Cat. No. G891; Applied Biological Materials Inc., Richmond, BC, Canada). Gene-specific primers were used at a final concentration of 0.5 μM, and β-actin was used as the reference gene. The primer sequences used for RT-qPCR analysis are listed in Table 1. Relative gene-expression levels were calculated using the ΔΔCt method, as previously described [43].

### 4.7. Western Blot Analysis

Protein expression and phosphorylation levels were analyzed by Western blotting as previously reported [12,44]. Briefly, total cellular proteins were extracted, separated by SDS-PAGE, and transferred onto nitrocellulose membranes. Membranes were incubated with specific primary antibodies against vimentin (Cat. No. 3295), N-cadherin (Cat. No. 4061), E-cadherin (Cat. No. 3195), phospho-ERK1/2 (Thr202/Tyr204; Cat. No. 4370), ERK1/2 (Cat. No. 4695), phospho-AKT (Ser473; Cat. No. 4060), AKT (Cat. No. 9272), phospho-NF-κB p65 (Ser536; Cat. No. 3033), and NF-κB p65 (Cat. No. 8242), all purchased from Cell Signaling Technology (Danvers, MA, USA); fibronectin (Cat. No. 15613-1-AP; Proteintech Group, Rosemont, IL, USA); and β-actin (Cat. No. 500-4554; Invitrogen™, Thermo Fisher Scientific, Waltham, MA, USA). Following incubation with the appropriate HRP-conjugated secondary antibodies, immunoreactive bands were detected by chemiluminescence and visualized using the ChemiDoc™ XRS+ imaging system with Image Lab™ software 5.0 (Bio-Rad Laboratories, Hercules, CA, USA).

### 4.8. Genome-Wide DNA Methylation Profiling

Genome-wide DNA methylation analysis was performed using eight independent biological samples, including three Ctrl samples, three samples treated with 100 μg/mL MO, and two samples treated with 200 μg/mL MO.

DNA concentration and purity were assessed using a Qubit 4 Fluorometer (Invitrogen™, Thermo Fisher Scientific, Waltham, MA, USA) and a NanoDrop One spectrophotometer (Thermo Fisher Scientific, Waltham, MA, USA), respectively. DNA quality was assessed using a 4200 TapeStation system (Agilent Technologies, Santa Clara, CA, USA). Bisulfite-converted DNA (250 ng) was used for genome-wide DNA methylation profiling using the Infinium MethylationEPIC v2.0 kit (Illumina, San Diego, CA, USA), which contains ~930K unique methylation sites in the most biologically significant regions of the human methylome. Briefly, bisulfite-converted DNA underwent whole-genome amplification for 20 h, followed by fragmentation. Fragmented DNA was precipitated, denatured, and hybridized to the BeadChips for 20 h at 48 °C. The BeadChips were then washed, and the hybridized primers were extended and labeled before scanning with the Illumina iScan system. Following data generation, raw intensity files (.idat) were imported into the R statistical environment for processing. Quality control, exploratory data analysis, and functional normalization were performed using the Bioconductor package minfi (version 1.56.0) [45]. The quality of the DNA methylation data was evaluated by calculating detection *p*-values for each CpG site across all samples. These *p*-values indicate whether the measured signal can be distinguished from background noise. Overall sample quality was assessed by calculating the mean detection *p*-value for each sample, with higher values indicating poorer signal detection. Functional normalization was performed using the preprocessFunnorm function to reduce technical variation in methylated and unmethylated signal intensities. Following normalization, the data were stored as a GenomicRatioSet object containing M-values (the log2 ratio of methylated to unmethylated intensities) for statistical analysis and Beta values for biological interpretation and visualization. Probes were filtered before downstream analysis to exclude unreliable signals and potential sources of confounding. Probes that failed the detection *p*-value cutoff of 0.01 in any sample were excluded. Probes containing common single-nucleotide polymorphisms (SNPs) at the CpG site or the single-base extension site were also removed to reduce potential confounding by genetic variation. Differentially methylated positions (DMPs) were identified by fitting linear models to M-values using the limma package (version 3.66.0) [46]. Differentially methylated regions (DMRs) spanning neighboring CpG sites were identified using the DMRcate package (version 3.6.0) [47]. The analysis was performed on an M-value matrix using the IlluminaHumanMethylationEPICv2anno.20a1.hg38 (version 1.0.1) annotation package and the IlluminaHumanMethylationEPICv2manifest (version 1.0.1) manifest package [48,49]. The numbers of hypermethylated and hypomethylated DMRs identified for each comparison are summarized in Supplementary Table S2. Over-representation analysis (ORA) of DMR-associated genes was performed using clusterProfiler (version 4.10.1) [50] and ReactomePA (version 1.54.0) [51] to identify enriched biological processes and pathways. For enrichment analyses, the background universe consisted of all genes associated with the DMPs included in the analysis, irrespective of their statistical significance. No additional correction was applied for differences in gene representation on the MethylationEPIC v2.0 array, including gene length, CpG density, or the number of CpG probes associated with individual genes. Thus, the use of this background universe restricts the enrichment analysis to genes represented in the analyzed methylation dataset but should not be considered a formal correction for array-specific probe-representation bias. Genes were mapped to Gene Ontology (GO) terms, KEGG pathways, and Reactome pathways. Enrichment was considered statistically significant at an FDR-adjusted *p*-value < 0.05.

### 4.9. Differential Methylation Analysis

Differentially methylated positions (DMPs) were identified using linear models applied to the M-value matrix with the limma package. *p*-values were adjusted for multiple testing using the Benjamini–Hochberg method, and CpGs with an adjusted *p*-value < 0.05 were considered significant. No minimum threshold for the absolute methylation difference (|Δβ|) was applied. The direction of differential methylation at individual CpG sites was determined by the sign of the limma logFC coefficient for each specified contrast.

Differentially methylated regions (DMRs) were identified using DMRcate with lambda = 1000, C = 2, and pcutoff = 0.05. DMRs with a Benjamini–Hochberg-adjusted *p*-value < 0.05 were considered significant. The direction of differential methylation for each DMR was determined by the sign of the mean methylation difference (meandiff) for each specified contrast.

The three comparisons were defined as 100 μg/mL MO relative to Ctrl, 200 μg/mL MO relative to Ctrl, and 100 μg/mL MO relative to 200 μg/mL MO. Accordingly, positive logFC or meandiff values indicate higher methylation at 100 μg/mL MO relative to Ctrl, at 200 μg/mL MO relative to Ctrl, and at 100 μg/mL MO relative to 200 μg/mL MO, respectively, whereas negative values indicate lower methylation in the corresponding comparisons.

Enrichment results were further processed and compared using Python 3.11. Figure 1, Figure 2, Figure 3, Figure 4, Figure 5, Figure 6, Figure 7, Figure 8, Figure 9, Figure 10, Figure 11, Figure 13, and Figures S2–S4 were generated using Matplotlib version 3.10.8 in Python version 3.12.14. The term “DNA methylation remodeling” used throughout the manuscript refers to experimentally detected changes in CpG methylation and DMR distribution. DMR-associated genes were used for pathway enrichment to provide biological interpretation of these changes; however, their genomic annotation and pathway association do not imply direct regulation of the corresponding genes or pathways, as the functional consequences of DNA methylation depend on genomic context, including promoter, enhancer, gene-body and intergenic localization.

### 4.10. Integrated Candidate-Gene Analysis

Representative candidate genes were selected from the integrated dataset based on their recurrence across significantly enriched functional categories and their biological relevance to processes associated with prostate cancer progression. Genes were selected for descriptive visualization, without formal assessment of network centrality or hub status.

To provide an integrated overview of the biological pathways associated with *Moringa oleifera* treatment, genes associated with significantly enriched GO Biological Process terms, KEGG pathways and Reactome pathways (FDR-adjusted *p* < 0.05) were combined for each comparison (100 μg/mL MO vs. Ctrl, 200 μg/mL MO vs. Ctrl, and 100 μg/mL MO vs. 200 μg/mL MO). For each comparison, genes were classified according to their occurrence in enriched gene sets associated with hypermethylated DMRs, hypomethylated DMRs, or both. Genes found exclusively in enriched gene sets associated with hypermethylated DMRs were classified as Hypermethylated, those found exclusively in enriched gene sets associated with hypomethylated DMRs as Hypomethylated, and those occurring in both as Both. Genes absent from the integrated enrichment analysis for a given comparison were classified as Not detected. These categories were used to generate the directional heat map shown in Figure 13, while the complete list of integrated candidate genes is provided in Table S1.

### 4.11. Statistical Analysis

RT-qPCR, Western blotting, and apoptosis data are presented as mean ± standard deviation (SD). Three independent biological experiments were performed, each with three technical replicates. Technical replicates were averaged within each biological experiment, and the independent biological experiments were used as the units of statistical analysis. Statistical analyses were performed using two-way analysis of variance (ANOVA) followed by Tukey’s multiple comparisons test. Adjusted *p*-values from Tukey’s post hoc test are reported for selected key comparisons in the corresponding figure legends. Statistical analyses were conducted using GraphPad Prism version 10.6.0 (GraphPad Software, Boston, MA, USA). Levels of significance are indicated as follows: * *p* < 0.05, ** *p* < 0.01 and *** *p* < 0.001.

### 4.12. Use of Generative Artificial Intelligence

Generative artificial intelligence (ChatGPT, OpenAI) was used during manuscript preparation to assist with language refinement, organization, and clarity of the text, and with the graphical revision of Figure 12. All experimental data, bioinformatic analyses, and scientific conclusions were generated and critically evaluated by the authors. All AI-assisted text and graphical content were reviewed, verified, and revised by the authors, who take full responsibility for the final content of the manuscript.

## 5. Conclusions

In conclusion, this study provides genome-wide evidence that *Moringa oleifera* treatment is associated with distinct DNA methylation changes at the two MO concentrations in PC3 cells, a highly aggressive metastatic prostate cancer cell line. DMR-associated genes were distributed across interconnected networks involving RHO/RAC GTPases, cell adhesion, extracellular-matrix organization, MAPK and PI3K-AKT signaling, angiogenesis, Wnt, NOTCH and EPH-Ephrin pathways. These methylation changes occurred in parallel with increased apoptosis at 200 μg/mL, reduced AKT and NF-κB phosphorylation, attenuation of EMT-related markers, and decreased expression of invasion-associated molecules. Importantly, the two MO concentrations were associated with distinct, bidirectional methylation profiles rather than a uniform increase or decrease in methylation with increasing concentration. Although the present data do not establish a direct causal relationship between individual methylation changes and downstream molecular responses, they identify a previously unexplored epigenomic response to *Moringa oleifera* in PC3 cells that differs between the two concentrations examined. Further integration of locus-specific methylation analysis with transcriptomic and functional studies will be required to define the biological consequences of these epigenetic changes.

## Figures and Tables

**Table 1:** Primer sequences for Real-Time Quantitative PCR (RT-qPCR) analyses.

Target	Primer Sequences (5′–3′)	Amplicon Length (bp)	GenBank Accession No.
*ALP*	Fw: CCGTGGCAACTCTATCTTTGG Rv: GCCATACAGGATGGCAGTGA	79	NM_000478.6
*BAX*	Fw: ACGAACTGGACAGTAACA Rv: TTGAAGTTGCCGTCAGAA	115	NM_138761.4
*BCL2*	Fw: ATGACTGAGTACCTGAAC Rv: AGGAGAAATCAAACAGAG	112	NM_000633.3
*c-MYC*	Fw: GGCGAACACACAACGTCTTGGAG Rv: GCTCAGGACATTTCTGTTAGAAG	298	NM_002467.6
*Cathepsin B*	Fw: CTACAGCGTCTCCAATAG Rv: GAAGTCCGAATACACAGA	91	L16510.1
*Cathepsin D*	Fw: TGCGACTGATACTATTAC Rv: AGATGAGATTATTGCCTAA	109	NM_001909.5
*Cystatin A*	Fw: AAGGGGACCTACATGTTCTGG Rv: ATAGGGCAGGGCTAAAAAGG	150	NM_005213.4
*Cystatin B*	Fw: GGGACAAACTACTTCATCAA Rv: GAGGGAGAGATTGGAACA	76	NM_000100.4
*Fibronectin*	Fw: CGAGGAGAGTGGAAGTGTGAGAG Rv: GGGTGAGGCTGCGGTTGG	108	NM_212482.1
*Integrin α2*	Fw: GTAGTTGACAACACAAAACAAACAA Rv: AAATAAAATTTTGTTGGAATGAAGC	150	NM_002203.3
*Integrin β1*	Fw: TGCCGGGTTTCACTTTGC Rv: GTGACATTGTCCATCATTTGGTAAA	70	NM_033668.1
*MMP2*	Fw: TGACGGTAAGGACGGACTC Rv: ATACTTCACACGGACCACTTG	124	NM_004530.2
*MMP9*	Fw: GGAGACCTGAGAACCAATC Rv: CCGAGTGTAACCATAGCG	72	NM_004994.3
*MMP13*	Fw: TTCCCAGTGGTGGTGATGAA Rv: CATGGAGCTTGCTGCATTCT	128	NM_002427.2
*E-Cadherin*	Fw: TGATTCTCTGCTCGTGTT Rv: CATTCGTTCAAGTAGTCATAGT	114	NM_004360.5
*N-Cadherin*	Fw: CATCATCATCCTGCTTATCCTTGT Rv: TTCTCCTCCACCTTCTTCATCA	148	M34064.1
*Occludin*	Fw: GCACCAAGCAATGACATA Rv: CAATAATGAGCATAGACAGGAT	154	U49184.1
*Vimentin*	Fw: GCTAACTACCAAGACACTATT Rv: TAGGTGGCAATCTCAATG	134	NM_003380.5
*β-actin*	Fw: ACCTTCTACAATGAGCTGCG Rv: TCCATCACGATGCCAGTGGTA	197	NM_001101.3

## Data Availability

The raw DNA methylation data and the corresponding processed data generated in this study have been deposited in the NCBI Gene Expression Omnibus (GEO) under accession number GSE348349. Additional data supporting the findings of this study are included in the article and its Supplementary Materials.

## Supplementary Materials

The following supporting information can be downloaded at: https://www.mdpi.com/article/10.3390/ijms27209144/s1.

## Abbreviations

AKT	Protein kinase B
ALP	Alkaline phosphatase
ANOVA	Analysis of variance
APC	Adenomatous polyposis coli
CCND2	Cyclin D2
CDH1	Cadherin 1 (E-cadherin)
CpG	Cytosine-phosphate-guanine
CRPC	Castration-resistant prostate cancer
DMR	Differentially methylated region
DMP	Differentially methylated position
DNA	Deoxyribonucleic acid
ECM	Extracellular matrix
EMT	Epithelial–mesenchymal transition
ERK	Extracellular signal-regulated kinase
FBS	Fetal bovine serum
FDR	False discovery rate
GC-MS	Gas chromatography-mass spectrometry
GO	Gene Ontology
GPCR	G protein-coupled receptor
GSTP1	Glutathione S-transferase Pi 1
HRP	Horseradish peroxidase
IGF-1	Insulin-like growth factor 1
IGF1R	Insulin-like growth factor 1 receptor
KEGG	Kyoto Encyclopedia of Genes and Genomes
MAPK	Mitogen-activated protein kinase
MMP	Matrix metalloproteinase
MO	*Moringa oleifera*
MSTFA	N-Methyl-N-(trimethylsilyl)trifluoroacetamide
c-MYC	MYC proto-oncogene, bHLH transcription factor
NF-κB	Nuclear factor kappa B
NOTCH3	Neurogenic locus notch homolog protein 3
NOTCH4	Neurogenic locus notch homolog protein 4
ORA	Over-representation analysis
PC3	Human prostate cancer cell line
PCa	Prostate cancer
PI3K	Phosphoinositide 3-kinase
PITX2	Paired-like homeodomain transcription factor 2
RARB	Retinoic acid receptor beta
RASSF1A	Ras association domain family member 1 isoform A
RT-qPCR	Reverse transcription quantitative polymerase chain reaction
RUNX3	Runt-related transcription factor 3
SD	Standard deviation
SDS-PAGE	Sodium dodecyl sulfate-polyacrylamide gel electrophoresis
TGF-β	Transforming growth factor beta
VEGF	Vascular endothelial growth factor
VEGFR2	Vascular endothelial growth factor receptor 2
