# Supplementary material for: Concentration-Associated DNA Methylation Remodeling of Oncogenic Signaling Networks Following Moringa oleifera Treatment in Metastatic PC3 Prostate Cancer Cells
Source: Int J Mol Sci. 2026 Oct 15;27(20):9144. doi: 10.3390/ijms27209144 (PMC13705501; doi:10.3390/ijms27209144)
Supplement: Supplementary file 1 [file ijms-27-09144-s001.zip › Supplementary.pdf]

Supplementary figures

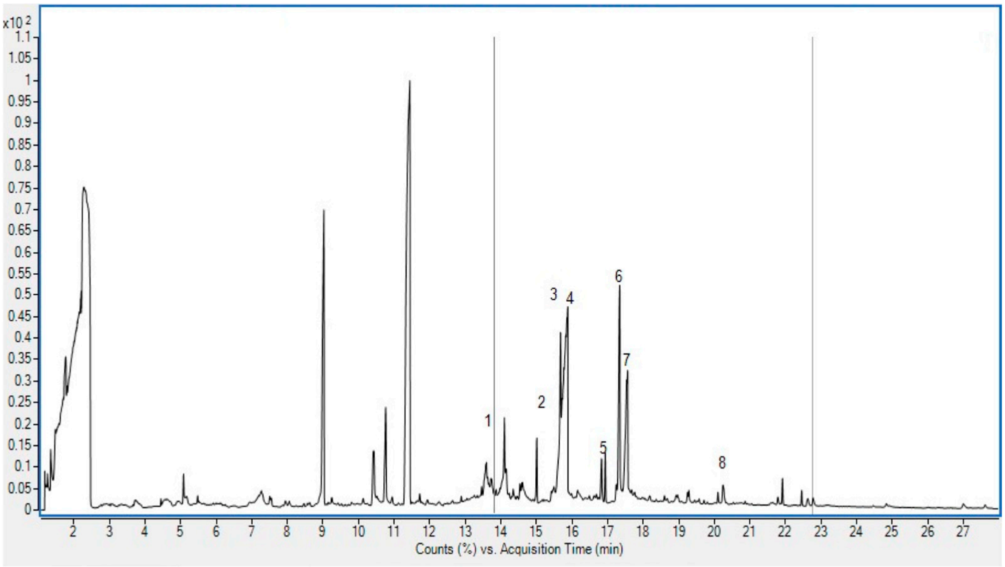

Figure S1. **Chromatogram of *Moringa oleifera* solution.** Representative GC-MS total ion chromatogram (TIC) of the *Moringa oleifera* leaf ethanolic extract after derivatization with MSTFA (full-scan acquisition, 41-500 m/z). Reproduced from [12] under CC BY 4.0. Numbered peaks correspond to the major compounds identified by NIST library matching: (1) tetradecanoic acid; (2) hexadecanoic acid, methyl ester (methyl palmitate); (3) n-hexadecanoic acid (palmitic acid); (4) isopropyl palmitate; (5) methyl stearate; (6) 9-octadecenoic acid (Z)-, methyl ester (methyl oleate); (7) octadecanoic acid (stearic acid); and (8) glycerol 1-palmitate (1-monopalmitin). Relative abundances of the identified compounds were reported in our previous study [12] under CC BY 4.0.

Reactome | MO100 vs Ctrl

A. Hypermethylated DMRs

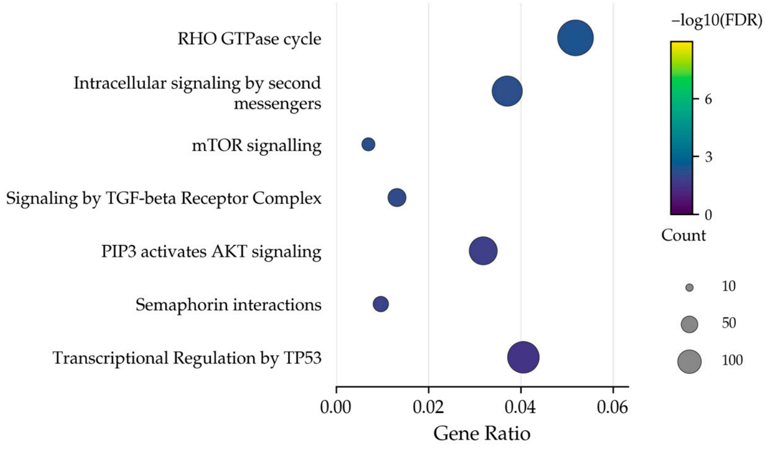

B. Hypomethylated DMRs

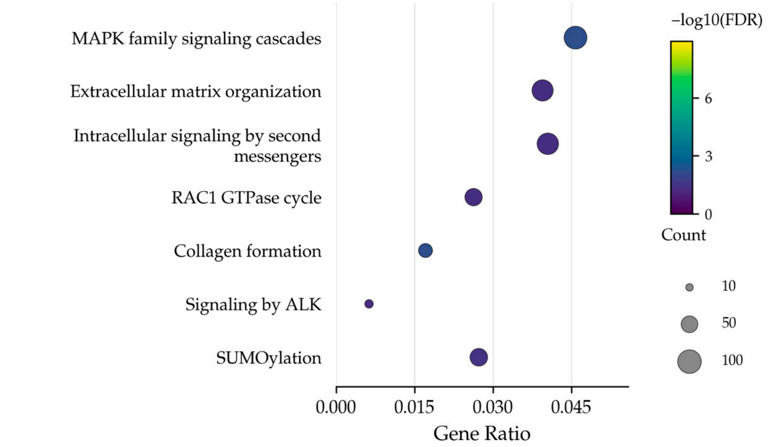

Figure S2. Reactome pathway enrichment analysis of DMR-associated genes in PC3 cells treated with 100 μg/mL Moringa oleifera extract compared with untreated controls. Selected significantly enriched pathways are shown for genes associated with hypermethylated DMRs (A; higher methylation in MO100 relative to Ctrl) and hypomethylated DMRs (B; lower methylation in MO100 relative to Ctrl). The x-axis shows the Gene Ratio, dot size represents the number of DMR-associated genes contributing to each pathway (Count), and dot color indicates −log10(FDR-adjusted p-value). Higher color-scale values indicate stronger statistical enrichment. Identical color and dot-area scales are used throughout all enrichment plots in the main text and Supplementary Materials. MO100 denotes treatment with 100 μg/mL MO; Ctrl denotes untreated cells.

Reactome | MO200 vs Ctrl

A. Hypermethylated DMRs

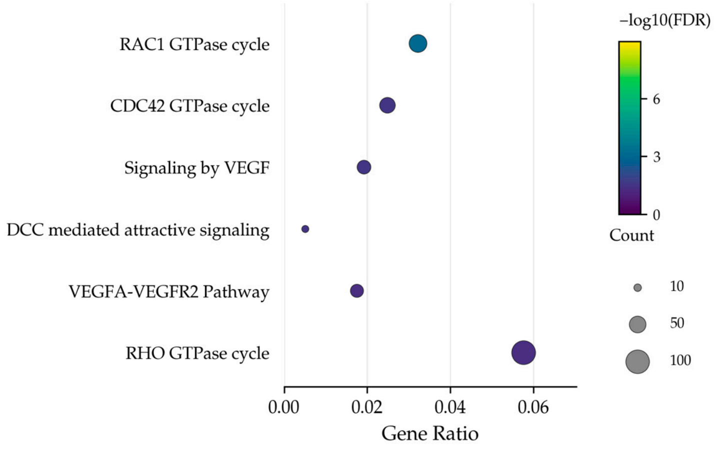

B. Hypomethylated DMRs

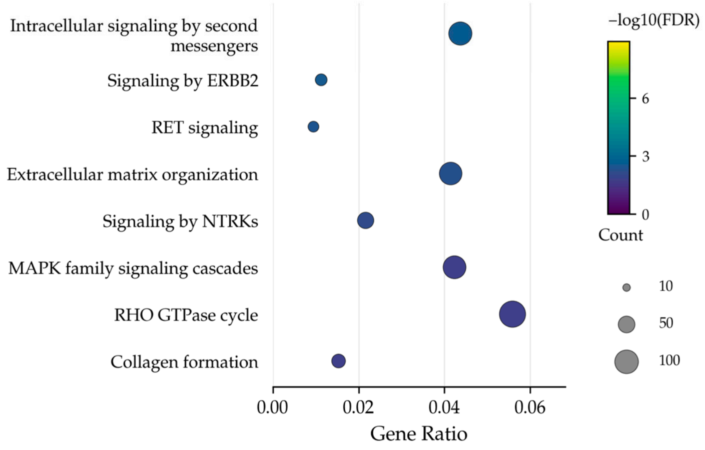

Figure S3. Reactome pathway enrichment analysis of DMR-associated genes in PC3 cells treated with 200 μg/mL Moringa oleifera extract compared with untreated controls. Selected significantly enriched pathways are shown for genes associated with hypermethylated DMRs (A; higher methylation in MO200 relative to Ctrl) and hypomethylated DMRs (B; lower methylation in MO200 relative to Ctrl). The x-axis shows the Gene Ratio, dot size represents the number of DMR-associated genes contributing to each pathway (Count), and dot color indicates −log10(FDR-adjusted p-value). Higher color-scale values indicate stronger statistical enrichment. Identical color and dot-area scales are used throughout all enrichment plots in the main text and Supplementary Materials. MO200 denotes treatment with 200 μg/mL MO; Ctrl denotes untreated cells.

Reactome | MO100 vs MO200

A. Hypermethylated DMRs

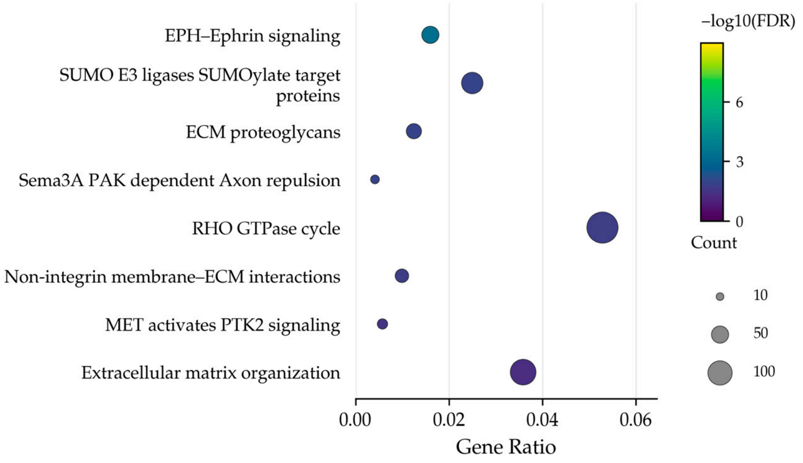

B. Hypomethylated DMRs

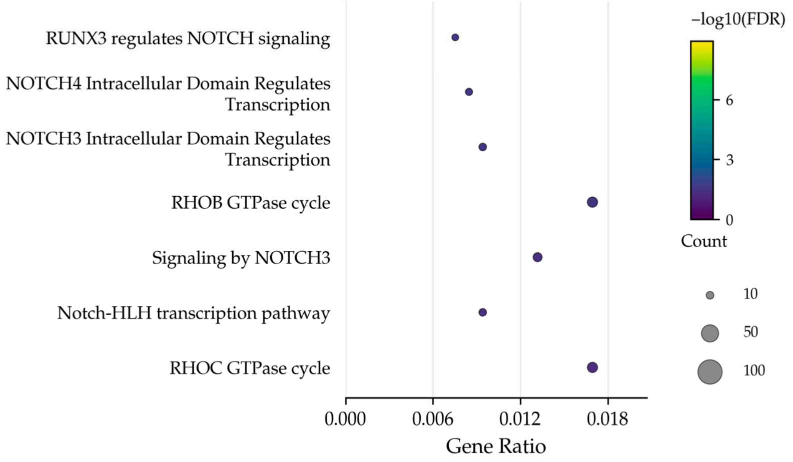

Figure S4. Reactome pathway enrichment analysis of DMR-associated genes in the direct comparison between PC3 cells treated with 100 and 200 µg/mL Moringa oleifera extract. Selected significantly enriched pathways are shown for genes associated with hypermethylated DMRs (A; higher methylation at 100 µg/mL relative to 200 µg/mL) and hypomethylated DMRs (B; lower methylation at 100 µg/mL relative to 200 µg/mL). The x-axis shows the Gene Ratio, dot size represents the number of DMR-associated genes contributing to each pathway (Count), and dot color indicates −log10(FDR-adjusted p-value). Higher color-scale values indicate stronger statistical enrichment. Identical color and dot-area scales are used throughout all enrichment plots in the main text and Supplementary Materials. MO100 denotes treatment with 100 µg/mL; MO200 denotes treatment with 200 µg/mL.
