# Supplementary material for: Concentration-Associated DNA Methylation Remodeling of Oncogenic Signaling Networks Following Moringa oleifera Treatment in Metastatic PC3 Prostate Cancer Cells
Source: Int J Mol Sci. 2026 Oct 15;27(20):9144. doi: 10.3390/ijms27209144 (PMC13705501; doi:10.3390/ijms27209144)
Supplement: Supplementary file 1 [file ijms-27-09144-s001.zip › Table S1.pdf]

Table S1. Complete list of the 86 candidate genes identified by integrating significantly enriched Gene Ontology (GO), KEGG, and Reactome terms and pathways. For each comparison (100 µg/mL MO vs Ctrl, 200 µg/mL MO vs Ctrl, and 100 µg/mL MO vs 200 µg/mL MO), genes were classified according to their representation in enriched gene sets associated with hypermethylated DMRs (Hyper), hypomethylated DMRs (Hypo), or both (Both). "Not detected" indicates that the gene was not represented in these enriched gene sets for the corresponding comparison. For the direct comparison between the two MO concentrations, Hyper indicates higher methylation at 100 µg/mL relative to 200 µg/mL, whereas Hypo indicates lower methylation at 100 µg/mL relative to 200 µg/mL. Checkmarks indicate the enrichment resources in which each gene was represented; blank cells indicate absence from the corresponding enriched gene sets. The number of enrichment resources indicates how many of the three resources supported the inclusion of each gene. Functional modules were assigned according to the predominant biological process or signaling pathway associated with each gene.

GENE	FUNCTIONAL MODULE	100 µg/mL MO VS CTRL	200 µg/mL MO VS CTRL	100 µg/mL MO VS 200 µg/mL MO	GO	KEGG	REACTOME	NUMBER OF ENRICHMENT RESOURCES
**RAC1**	RHO/RAC GTPase signaling	Hyper	Hyper	Hyper	✔	✔	✔	3
**RHOA**	RHO/RAC GTPase signaling	Hyper	Both	Not detected	✔	✔	✔	3
**ROCK1**	RHO/RAC GTPase signaling	Hyper	Hyper	Hyper	✔	✔	✔	3
**ROCK2**	RHO/RAC GTPase signaling	Hyper	Hyper	Both	✔	✔	✔	3
**DLC1**	RHO/RAC GTPase signaling	Hyper	Hyper	Hypo	✔		✔	2
**TIAM1**	RHO/RAC GTPase signaling	Not detected	Hyper	Hypo	✔	✔	✔	3
**DAAM1**	RHO/RAC GTPase signaling	Hyper	Both	Both	✔	✔	✔	3
**PAK1**	RHO/RAC GTPase signaling	Hyper	Not detected	Hyper	✔	✔	✔	3
**PAK3**	RHO/RAC GTPase signaling	Hyper	Both	Hyper	✔	✔	✔	3
**PAK4**	RHO/RAC GTPase signaling	Hyper	Hypo	Hyper	✔	✔	✔	3
**ARHGEF7**	RHO/RAC GTPase signaling	Both	Both	Hyper	✔	✔	✔	3

**VAV2**	RHO/RAC GTPase signaling	Both	Hypo	Hyper	✔	✔	✔	3
**VAV3**	RHO/RAC GTPase signaling	Hyper	Hyper	Hyper	✔	✔	✔	3
**AKT1**	PI3K-AKT-mTOR	Hyper	Both	Both	✔	✔	✔	3
**AKT2**	PI3K-AKT-mTOR	Both	Hyper	Hyper	✔	✔	✔	3
**AKT3**	PI3K-AKT-mTOR	Not detected	Both	Not detected	✔	✔	✔	3
**MTOR**	PI3K-AKT-mTOR	Hyper	Both	Both	✔	✔	✔	3
**PIK3CA**	PI3K-AKT-mTOR	Hypo	Hypo	Not detected	✔	✔	✔	3
**PIK3CB**	PI3K-AKT-mTOR	Both	Hyper	Hypo	✔	✔	✔	3
**PIK3R1**	PI3K-AKT-mTOR	Not detected	Hypo	Not detected	✔	✔	✔	3
**PIK3R2**	PI3K-AKT-mTOR	Hypo	Hypo	Hyper	✔	✔	✔	3
**RICTOR**	PI3K-AKT-mTOR	Hyper	Hypo	Hyper	✔	✔	✔	3
**RPTOR**	PI3K-AKT-mTOR	Both	Hypo	Both	✔	✔	✔	3
**EGFR**	RTK/MAPK	Both	Both	Both	✔	✔	✔	3
**ERBB2**	RTK/MAPK	Both	Hypo	Hyper	✔	✔	✔	3
**ERBB3**	RTK/MAPK	Hypo	Hyper	Hyper	✔	✔	✔	3
**ERBB4**	RTK/MAPK	Not detected	Hypo	Not detected	✔	✔	✔	3
**MET**	RTK/MAPK	Both	Hyper	Hyper	✔	✔	✔	3
**FGFR1**	RTK/MAPK	Both	Hypo	Not detected	✔	✔	✔	3
**FGFR4**	RTK/MAPK	Hyper	Both	Hyper	✔	✔	✔	3
**RET**	RTK/MAPK	Hyper	Hypo	Hyper	✔	✔	✔	3
**SRC**	RTK/MAPK	Both	Hypo	Hyper	✔	✔	✔	3
**FYN**	RTK/MAPK	Both	Both	Hyper	✔	✔	✔	3
**HRAS**	RTK/MAPK	Not detected	Hypo	Hyper	✔	✔	✔	3
**BRAF**	RTK/MAPK	Not detected	Hypo	Not detected	✔	✔	✔	3
**MAP2K2**	RTK/MAPK	Not detected	Hypo	Not detected	✔	✔	✔	3
**MAPK14**	RTK/MAPK	Hypo	Hypo	Hyper	✔	✔	✔	3
**FN1**	ECM organization / cell adhesion	Hypo	Not detected	Hyper	✔	✔	✔	3
**ITGB1**	ECM organization / cell adhesion	Both	Not detected	Hyper	✔	✔	✔	3

**PTK2**	ECM organization / cell adhesion	Hyper	Not detected	Hyper	✔	✔	✔	3
**PTK2B**	ECM organization / cell adhesion	Both	Hypo	Both	✔	✔	✔	3
**PXN**	ECM organization / cell adhesion	Not detected	Hyper	Hyper	✔	✔	✔	3
**CAV1**	ECM organization / cell adhesion	Hyper	Hyper	Hyper	✔	✔	✔	3
**THBS1**	ECM organization / cell adhesion	Hyper	Both	Hyper	✔	✔	✔	3
**COL1A1**	ECM organization / cell adhesion	Not detected	Not detected	Hyper	✔	✔	✔	3
**COL1A2**	ECM organization / cell adhesion	Hypo	Hypo	Hypo	✔	✔	✔	3
**COL3A1**	ECM organization / cell adhesion	Not detected	Hypo	Hyper	✔	✔	✔	3
**COL4A1**	ECM organization / cell adhesion	Both	Hyper	Both	✔	✔	✔	3
**COL4A2**	ECM organization / cell adhesion	Both	Both	Not detected	✔	✔	✔	3
**COL5A1**	ECM organization / cell adhesion	Both	Hypo	Hyper	✔	✔	✔	3
**LAMB1**	ECM organization / cell adhesion	Not detected	Hypo	Not detected	✔	✔	✔	3
**VCL**	ECM organization / cell adhesion	Hyper	Not detected	Hyper	✔	✔	✔	3
**TLN1**	ECM organization / cell adhesion	Hyper	Hypo	Hyper	✔	✔	✔	3
**VEGFC**	VEGF / angiogenesis	Not detected	Hyper	Not detected	✔	✔	✔	3
**FLT1**	VEGF / angiogenesis	Both	Both	Hypo	✔	✔	✔	3
**KDR**	VEGF / angiogenesis	Hyper	Not detected	Not detected	✔	✔	✔	3
**NRP1**	VEGF / angiogenesis	Hyper	Hyper	Not detected	✔	✔	✔	3
**TGFB1**	TGF-β / Wnt	Both	Hyper	Hyper	✔	✔	✔	3
**TGFB2**	TGF-β / Wnt	Not detected	Hypo	Hyper	✔	✔	✔	3
**TGFB3**	TGF-β / Wnt	Hyper	Hyper	Hyper	✔	✔	✔	3
**SMAD3**	TGF-β / Wnt	Hyper	Hyper	Both	✔	✔	✔	3
**SMAD4**	TGF-β / Wnt	Hyper	Hypo	Hyper	✔	✔	✔	3

**CTNNB1**	TGF-β / Wnt	Not detected	Hyper	Not detected	✔	✔	✔	3
**TGFBR1**	TGF-β / Wnt	Not detected	Hypo	Not detected	✔	✔	✔	3
**AXIN1**	TGF-β / Wnt	Hyper	Hypo	Hyper	✔	✔	✔	3
**LRP5**	TGF-β / Wnt	Hyper	Hypo	Hyper	✔	✔	✔	3
**NOTCH1**	NOTCH	Both	Hypo	Hyper	✔	✔	✔	3
**NOTCH3**	NOTCH	Hyper	Hypo	Hypo	✔	✔	✔	3
**JAG1**	NOTCH	Hypo	Not detected	Both	✔		✔	2
**RBPJ**	NOTCH	Hyper	Both	Both	✔	✔	✔	3
**MAML1**	NOTCH	Hypo	Not detected	Both	✔		✔	2
**MAML2**	NOTCH	Not detected	Hypo	Not detected	✔		✔	2
**MAML3**	NOTCH	Both	Hypo	Both	✔		✔	2
**CREBBP**	NOTCH	Both	Hypo	Both	✔	✔	✔	3
**EP300**	NOTCH	Both	Not detected	Both	✔	✔	✔	3
**EPHA2**	EPH-Ephrin	Hyper	Not detected	Hyper	✔	✔	✔	3
**EPHA4**	EPH-Ephrin	Hypo	Not detected	Hyper	✔	✔	✔	3
**EPHA5**	EPH-Ephrin	Not detected	Not detected	Hyper	✔	✔	✔	3
**EPHA6**	EPH-Ephrin	Both	Not detected	Hyper	✔	✔	✔	3
**EPHA8**	EPH-Ephrin	Not detected	Hypo	Hyper	✔	✔	✔	3
**EPHB1**	EPH-Ephrin	Both	Hypo	Both	✔	✔	✔	3
**EPHB2**	EPH-Ephrin	Both	Not detected	Hyper	✔	✔	✔	3
**EPHB4**	EPH-Ephrin	Hypo	Not detected	Hyper	✔	✔	✔	3
**EFNA1**	EPH-Ephrin	Hyper	Hypo	Hyper	✔	✔	✔	3
**EFNA3**	EPH-Ephrin	Not detected	Hypo	Hyper	✔	✔	✔	3
**EFNA5**	EPH-Ephrin	Hyper	Not detected	Hyper	✔	✔	✔	3
