# Supplementary material for: Concentration-Associated DNA Methylation Remodeling of Oncogenic Signaling Networks Following Moringa oleifera Treatment in Metastatic PC3 Prostate Cancer Cells
Source: Int J Mol Sci. 2026 Oct 15;27(20):9144. doi: 10.3390/ijms27209144 (PMC13705501; doi:10.3390/ijms27209144)
Supplement: Supplementary file 1 [file ijms-27-09144-s001.zip › Table S2.pdf]

Table S2. Summary of differentially methylated regions (DMRs) identified in each comparison. DMRs were identified using DMRcate (v3.6.0; lambda = 1000, C = 2, pcutoff = 0.05). DMR direction was determined according to the mean methylation difference (meandiff), with positive values indicating hypermethylation and negative values indicating hypomethylation in the first condition relative to the reference condition. For the direct comparison between the two MO concentrations, hypermethylation therefore indicates higher methylation at 100 µg/mL MO relative to 200 µg/mL MO, whereas hypomethylation indicates lower methylation at 100 µg/mL MO relative to 200 µg/mL MO.

Comparison	*Hypermethylated DMRs, n*	*Hypomethylated DMRs, n*	*Total DMRs, n*
100 µg/mL MO vs Ctrl	**12,436**	**5,234**	**17,670**
200 µg/mL MO vs Ctrl	**4,397**	**5,608**	**10,005**
100 µg/mL MO vs 200 µg/mL MO	**8,487**	**2,797**	**11,284**
